# Determinants of COVID-19 vaccine fatigue

**DOI:** 10.1038/s41591-023-02282-y

**Published:** 2023-03-27

**Authors:** Tanja A. Stamm, Julia Partheymüller, Erika Mosor, Valentin Ritschl, Sylvia Kritzinger, Alessia Alunno, Jakob-Moritz Eberl

**Affiliations:** 1 Institute of Outcomes Research, Center for Medical Data Science, Medical University of Vienna, Vienna, Austria; 2 Ludwig Boltzmann Institute for Arthritis and Rehabilitation, Vienna, Austria; 3 Department of Government, University of Vienna, Vienna, Austria; 4 Department of Life, Health & Environmental Sciences, University of L’Aquila and Internal Medicine and Nephrology Division, ASL1 Avezzano-Sulmona-L’Aquila, San Salvatore Hospital, L’Aquila, Italy; 5 Department of Communication, University of Vienna, Vienna, Austria

**Keywords:** Health policy, Interdisciplinary studies

## Abstract

There is growing concern that Coronavirus Disease 2019 (COVID-19) vaccine fatigue will be a major obstacle in maintaining immunity in the general population. In this study, we assessed vaccine acceptance in future scenarios in two conjoint experiments, investigating determinants such as new vaccines, communication, costs/incentives and legal rules. The experiments were embedded in an online survey (*n* = 6,357 participants) conducted in two European countries (Austria and Italy). Our results suggest that vaccination campaigns should be tailored to subgroups based on their vaccination status. Among the unvaccinated, campaign messages conveying community spirit had a positive effect (0.343, confidence interval (CI) 0.019–0.666), whereas offering positive incentives, such as a cash reward (0.722, CI 0.429–1.014) or voucher (0.670, CI 0.373–0.967), was pivotal to the decision-making of those vaccinated once or twice. Among the triple vaccinated, vaccination readiness increased when adapted vaccines were offered (0.279, CI 0.182–0.377), but costs (−0.795, CI −0.935 to −0.654) and medical dissensus (−0.161, CI −0.293 to −0.030) reduced their likelihood to get vaccinated. We conclude that failing to mobilize the triple vaccinated is likely to result in booster vaccination rates falling short of expectations. For long-term success, measures fostering institutional trust should be considered. These results provide guidance to those responsible for future COVID-19 vaccination campaigns.

## Abstract (web summary)

Findings from two conjoint experiments conducted in Austria and Italy revealed differences in the determinants of COVID-19 vaccine hesitancy and vaccine fatigue, suggesting that vaccination campaigns should be tailored to different groups according to previous vaccination status.

## Main

Vaccines are likely to remain one of the essential tools to fight the severe acute respiratory syndrome coronavirus-2 (SARS-CoV-2) pandemic^1^. Vaccines against Coronavirus Disease 2019 (COVID-19) are now widely available in many countries, and, since the initial rollout, progress in vaccine development has been made. Notably, new adapted vaccines targeting specific virus variants have come into use, and additional non-mRNA vaccines have achieved regulatory approval, with more than 200 additional vaccine candidates currently under development^1^.

Vaccines, however, can be effective only if people get vaccinated. Unfortunately, several behavioral factors threaten to undercut the advances in vaccine supply and development^2,3^. Previous research has focused, in particular, on COVID-19 vaccine hesitancy as an obstacle to primary vaccinations, which have plateaued and come to a halt over time^4–8^. In addition, the enthusiasm for booster vaccinations has decreased among the already vaccinated, and ‘vaccine fatigue’ (also known as ‘booster fatigue’, ‘booster hesitancy’ or ‘immunization fatigue’)^9–11^ has emerged as a growing concern for public health officials. The concept of vaccine fatigue is already known from the influenza context, where suboptimal uptake has repeatedly resulted in many unnecessary deaths^12^. Evolving evidence suggests that, like in other vaccination regimens^12^, regular or seasonal booster vaccinations against COVID-19 could be necessary to counteract waning immunity and offer protection against newly emerging virus variants^13–17^. Although global expert consensus on booster recommendations for COVID-19 is still emerging, it is very likely that the failure to address vaccination hesitancy and fatigue could have serious public health consequences in the long run and, in turn, increase pressure on healthcare systems.

To prevent such negative outcomes, many national governments and public health experts have sought to develop vaccination and communication strategies for the medium-term future, considering the contingencies that arise from the unknown course of evolution of the virus^18–20^. Previous research on vaccine fatigue in the influenza context suggested that some measures, such as written reminders^21^, could also be effective in the context of COVID-19. However, we do not know to what extent these results apply to COVID-19, as the heterogeneous immunization status in the population and its underlying psychology need to be taken into account to design effective messages and communication strategies. Previous research on COVID-19 vaccination was conducted in the context of the initial rollout, with circumstances no longer present (for example, shortage of supply and vaccine envy) and has, thus, primarily addressed the concept of vaccine hesitancy and, only to a much smaller extent, vaccine fatigue. In addition, previous survey experiments focused on one single aspect (for example, the role of incentives)^22^, but, in a situation where there are many unknown contextual features, a simultaneous assessment of various factors is necessary to account for the possible contingencies of future scenarios (for example, the severity of new virus variants). We, thus, need to know more about COVID-19 vaccine fatigue, possible interventions to foster the acceptance of boosters and possible contextual contingencies.

With that in mind, two practically and theoretically relevant research questions emerge that we address in this paper. (1) Should vaccination campaigns adopt similar or different strategies for primary and booster vaccinations? (2) What are the most relevant contextual features and the most effective interventions that may affect vaccine acceptance in future scenarios? Hence, the overarching aim of this study was to provide evidence for designing effective vaccination campaigns, taking into account both the heterogeneous immunization status in the population as well as possible contextual contingencies.

To do so, we designed two conjoint experiments portraying possible future scenarios and assessing their impact on vaccination intentions and related attitudes. Conjoint experiments^23^ are highly suitable to model the likely outcomes of alternative future scenarios as they allow researchers to manipulate multiple attributes of a hypothetical scenario and measure the responses of participants considering all attributes jointly. To identify relevant attributes, we reviewed the literature on COVID-19 vaccine acceptance. The literature review revealed that properties of vaccines^24–26^, communication (for example, campaign messages^27–30^, expert consensus^31^ and celebrity endorsement^32–34^), costs/incentives^8,34–36^ and legal rules (for example, vaccine passports^36,37^ and vaccine mandates^38,39^) may matter most for COVID-19 vaccine uptake. We transferred those attributes to the current context, where new vaccines have become available and there was uncertainty about some future conditions (such as virus variants), and we evaluated the relevance of the various attributes simultaneously for different subgroups based on their immunization status. By doing so, the study aimed to increase understanding of how vaccine hesitancy and vaccine fatigue could be most effectively addressed.

## Results

### Background characteristics of the study population

The conjoint experiments of our study were embedded in a cross-sectional online survey conducted in two European countries, Austria and Italy, simultaneously between 19 July and 8 August 2022. Like many other developed nations, such as the United Kingdom, France, the United States and Canada^2,3,40^, both countries had been experiencing stagnating vaccination rates. By August 2022, 77.1% of Austrians had completed the primary course of vaccinations, and 59.2% had received three doses of the vaccine^41^, whereas, in Italy, both primary vaccinations (80.2%) and booster vaccinations (71.5%) were more common^42^. The target population of the survey were residents aged 14 years and older. Recommendations regarding second boosters (after an initial course of two vaccinations and a booster) were still unknown at the time of the fieldwork, but it appeared plausible that second boosters would be offered to everyone eligible for this study in the fall, which, as of December 2022, is, in fact, the case according to the national guidelines^43,44^. Both the Austrian and Italian samples matched the target quotas regarding gender, age groups, regions and education. Overall, 6,357 respondents took part in the survey (Austria: *n* = 3,187; Italy: *n* = 3,170). Further details on the survey and the countries are provided in the Methods section.

**Extended Data Fig. 1:**
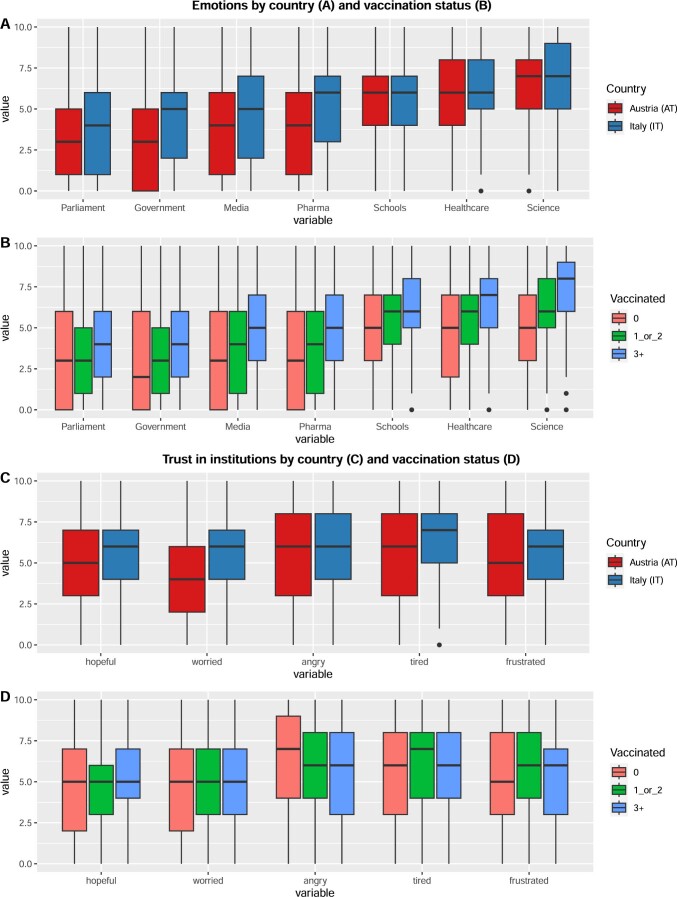
Emotions by country (A) and vaccination status (B), as well as trust in institutions by country (C) and vaccination status (D). To assess emotions, respondents were asked the following question: ‘When you think of the Corona crisis, how much do you feel...?’. For each item (hopeful, worried, angry, tired, and frustrated), the participants recorded their responses on a scale ranging from 0 ‘not at all’ to 10 ‘very much’. In total, 6,357 respondents participated in the survey (Austria: n = 3,187; Italy: n = 3,170); 270 (4%) participants did not answer the question about hope; 208 (3%) answers were missing for worry, 240 (4%) for anger, 236 (4%) for fatigue and 234 (4%) for frustration. To assess institutional trust, the respondents were asked, ‘How much trust do you have in each of the institutions mentioned?’. For each item (parliament, government, media, pharmaceutical industry, schools, healthcare system, and science), the participants recorded their responses on a 0-to-10 scale, ranging from 0 ‘no trust at all’ to 10 ‘very much trust’. 211 (3%) participants did not answer the question about parliament; 224 (4%) answers were missing for government, 207 (3%) for media, 238 (4%) for the pharmaceutical industry, 236 (4%) for schools, 186 (3%) for the health care system and 199 (3%) for science. Boxplots show medians +/-1.5 interquartile range (IQR). For information on the reliability and validity of the measurement, see the methods section.

**Extended Data Fig. 2:**
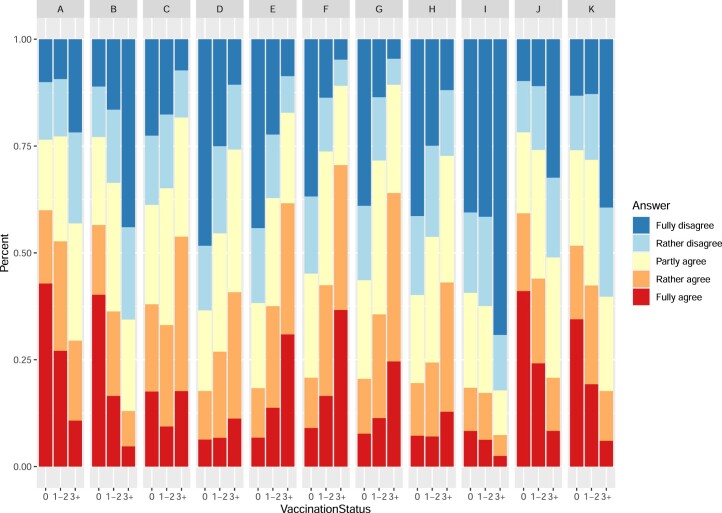
Attitudes toward COVID-19 vaccination by vaccination status. This bar chart shows the results of the item battery which we ran to assess attitudes towards the COVID-19 vaccination. Item (A) refers to ‘I am concerned about unanticipated side effects of the vaccination.’, (B) ‘I prefer to rely on my immune system rather than vaccination.’, (C) ‘I feel well informed about how vaccines work.’, (D) ‘Vaccination allows me to live as I did before the pandemic.’, (E) ‘Vaccination allows me to protect others.’, (F) ‘With a vaccination, I can protect myself.’, (G) ‘Vaccinations approved by the authorities are safe.’, (H) ‘Authorities provide sufficient information about how vaccines work.’, (I) ‘My everyday life is too stressful, I don't have time for vaccination.’, (J) ‘Vaccination does not help, you still get sick.’, and (K) ‘The current virus variant is mild, so I don't need vaccination.’ Bars depict the stacked proportions for each response category. In total, 6,357 respondents participated in the survey (Austria: n = 3,187; Italy: n = 3,170); missing values ranged between 6 to 8% for these items: 394 (6%) answers were missing for A, 413 (6%) for B, 406 (6%) for C, 387 (6%) for D, 422 (6%) for E, 429 (7%) for F, 536 (7%) for G, 418 (8%) for H, 529 (7%) for I, 433 (7%) for J, and 694 (11%) for K. For information on the measurement’s reliability and validity, see the methods section.

The readiness to get vaccinated was measured on a scale from 0 to 10 and was higher in Italy (5.8 ± 2.6) than in Austria (5.3 ± 3.3), averaging across all scenarios in both conjoint experiments. Respondents in both countries reported high levels of pandemic fatigue and showed low to medium levels of trust in parliament and government (Extended Data Fig. 1). Across both countries, 61% of respondents had already received three or more doses of a COVID-19 vaccine; 14% had received one or two doses; and 25% reported not being vaccinated. The triple-vaccinated group reported the highest likelihood to get vaccinated again across all scenarios, with a mean of 6.6 (±2.5); those with one or two doses showed intermediate likelihood, with a mean of 4.7 (±2.6); and the unvaccinated group was the least likely to declare vaccination readiness under any of the shown scenarios, with a mean of 3.4 (±3.0) (Supplementary File 3). The triple-vaccinated group differed from the other two groups, exhibiting higher mean age, levels of education and trust in institutions (Extended Data Fig. 1), which is in line with previous research that has also stressed that political factors, such as trust in the political system, matter as determinants of confidence in vaccines and vaccine uptake^45,46^. As might be expected, pandemic fatigue was highest among those with one or two vaccinations (Extended Data Fig. 1). Vaccination status also differed with regard to their underlying attitudes toward COVID-19 vaccination. The unvaccinated group was the most concerned about unforeseen side effects of the vaccine and least convinced of its benefits, whereas the triple-vaccinated group was most convinced of the benefits and least concerned about vaccine safety. Those with one or two vaccine doses were typically in between the unvaccinated and the triple vaccinated and felt the least well informed of all three groups (Extended Data Fig. 2).

### Effects of scenarios for a hypothetical vaccination campaign from experiment 1

**Fig. 1:**
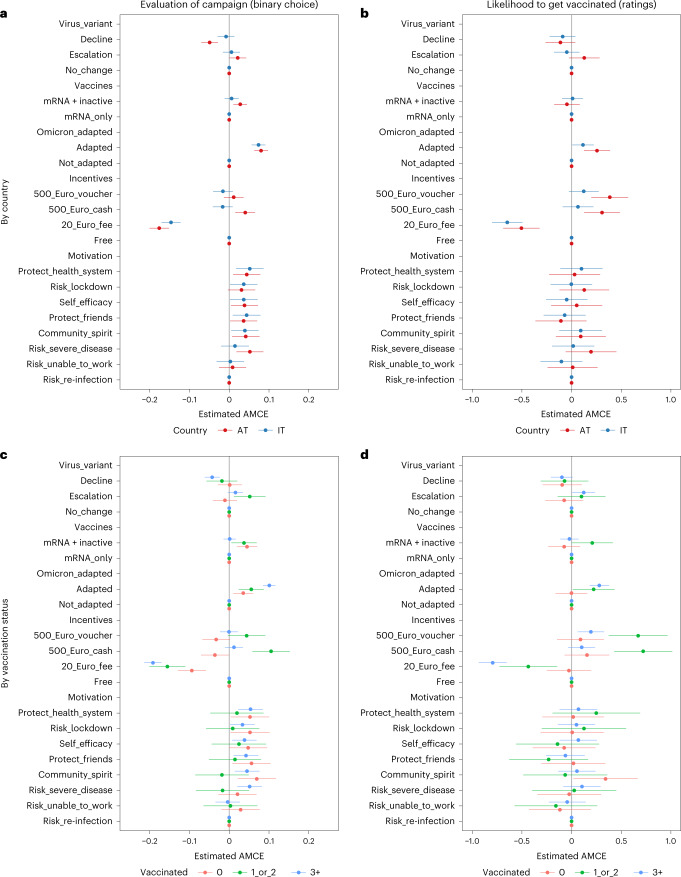
Effects of scenarios for a hypothetical vaccination campaign (experiment 1). **a**–**d**, The manipulated attributes in this experiment included the severity of the circulating virus variant (decline, escalation, no change; ‘Virus_variant’); the availability of the protein subunit COVID-19 vaccine Novavax or the inactivated whole virus vaccine Valneva in addition to mRNA vaccines (mRNA + inactive, mRNA only; ‘Vaccines’); Omicron adaption (adapted, not adapted; ‘Omicron_adapted’); costs/incentives (a voucher with a value of 500 Euros, cash of the same amount, a fee of 20 Euros for getting vaccinated and the vaccination being free of charge; ‘Incentives’); and motivational campaign messages (‘Motivation’) regarding the protection of the healthcare system (‘Protect_health_system’), the reduction of the risk for a lockdown (‘Risk_lockdown’), the ability to contribute oneself (‘Self_efficacy’), the protection of friends (‘Protect_friends’), the protection of the community (‘Community_spirit’), the reduction of the risk for severe disease (‘Risk_severe_disease’), the risk for being unable to work (‘Risk_unable_to_work’) and the risk of re-infection (‘Risk_re-infection’). We calculated AMCEs. Data are presented as AMCE estimates ± 95% CIs. Exact *P* values are shown in Supplementary Files 5–8. In total, 6,357 respondents participated in this experiment (Austria: *n* = 3,187; Italy: *n* = 3,170).

In experiment 1, we showed the respondents two alternate scenarios for a hypothetical vaccination campaign in the fall. We first asked the respondents to assess in which scenario they would evaluate the vaccination campaign more favorably (binary choice). Then, respondents were asked to rate their likelihood to get vaccinated for each scenario on a 0–10 scale (ratings). The manipulated attributes in this experiment (Fig. 1) included the severity of the circulating virus variant, the availability of the protein subunit COVID-19 vaccine Novavax or the inactivated whole virus vaccine Valneva, Omicron adaption, costs/incentives and campaign messages. For the full wording of the experimental treatments and outcome variables, see the Methods section and Supplementary File 3. The results are shown in Fig. 1.

We found limited evidence that more severe virus variants would result in a higher likelihood to get vaccinated. In both countries, most of the effects were not statistically significant. Only the triple-vaccinated group reported a marginally significantly higher likelihood to get vaccinated (0.122, confidence interval (CI) 0.007–0.236) when facing a more severe virus variant. In contrast, the effects were not statistically significant for those with one or two doses and for the unvaccinated in the escalation scenario. Once-vaccinated and twice-vaccinated respondents at least evaluated the vaccination campaign more favorably in the light of a more severe variant, which could imply a heightened receptiveness for campaigns under such circumstances.

Our results support the notion that new vaccines are likely to play a role in future campaigns. We found evidence for both countries (although stronger in Austria and only marginally significant in Italy) that variant-adapted (in our experiment, Omicron-adapted) vaccines could contribute to a greater readiness to get vaccinated (Austria: 0.256, CI 0.125–0.387; Italy: 0.037, CI 0.007–0.223), in particular among the triple vaccinated (0.279, CI 0.182–0.377). Also, vaccination campaigns were evaluated more positively when variant-adapted vaccines were advertised. The evidence in favor of an added value of non-mRNA vaccines, such as the protein subunit COVID-19 vaccine Novavax or the inactivated whole virus vaccine Valneva, was somewhat mixed. New vaccines of this type seemed to have the highest potential among those already vaccinated once or twice. This group reported a higher likelihood to get vaccinated (0.208, CI 0.000–0.416) that was only marginally significant. For the unvaccinated, we saw no change in vaccination intentions based on the availability of new vaccines, although they seemed to evaluate vaccination campaigns more favorably when additional new vaccines of either type were made available.

In line with previous research, our results confirm that costs and incentives are likely to matter for vaccination decisions in future scenarios. We found evidence in both countries that even minor costs (20 Euros) could strongly reduce vaccine uptake (Austria: −0.505, CI −0.688 to −0.322; Italy: −0.647, CI −0.801 to −0.494), in particular among the triple vaccinated (−0.795, CI −0.935 to −0.654). Increasing vaccine acceptance with positive incentives, such as vouchers or monetary rewards, was more challenging. Even with a fairly generous reward of 500 Euros, the effects remained conditional and country specific. In particular, those vaccinated already once and twice reacted most strongly to incentives and reported a higher likelihood to get vaccinated when offered positive incentives (cash: 0.722, CI 0.429–1.014; voucher: 0.670, CI 0.373–0.967). We also found that respondents from Austria were more susceptible to incentives (cash: 0.307, CI 0.127–0.487; voucher: 0.384, CI 0.199–0.570).

As the final component of experiment 1, we tested different motivational appeals as campaign messages. We mostly found no effects of motivational appeals on vaccination intentions, suggesting that most messages were similarly effective or ineffective. Among the unvaccinated and compared to the baseline message warning of the re-infection risk, however, emphasizing people’s sense of community (community spirit: 0.343, CI 0.019–0.666) may be able to increase vaccine uptake positively. Otherwise, the motivational appeals mainly affected how positively respondents evaluated the vaccination campaign, with most messages performing about equally well.

### Effects of scenarios of media communication about vaccinations from experiment 2

**Fig. 2:**
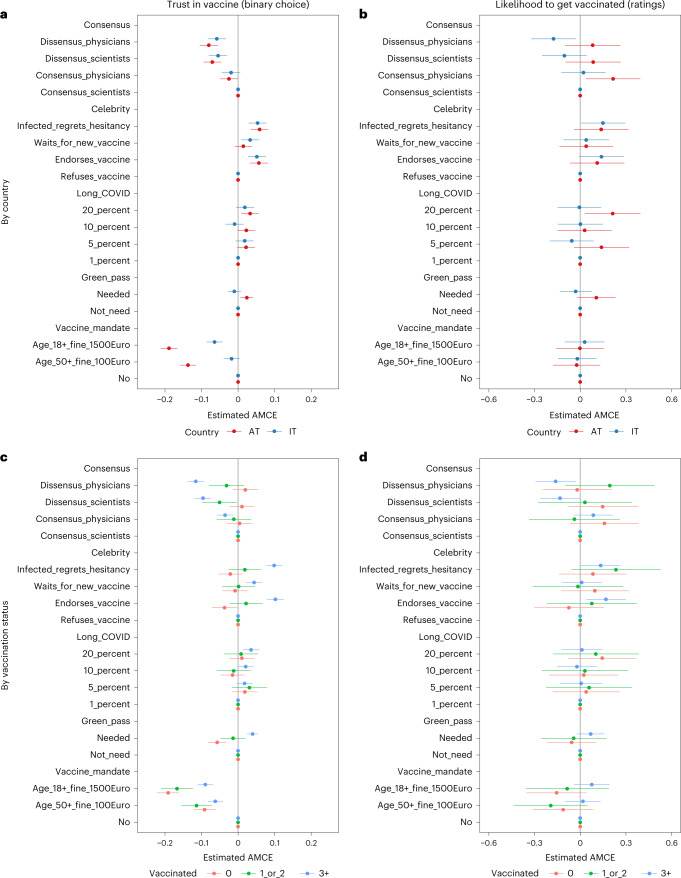
Effects of scenarios of media communication about vaccinations (experiment 2). **a**–**d**, The manipulated attributes of each media report included the consensus or dissensus of physicians or scientists (‘Consensus’); celebrities regretting their vaccination hesitancy when getting infected, waiting for a new vaccine, endorsing the vaccination or refusing the vaccination (‘Celebrity’); the likelihood chance to get Long COVID after infection, with rates ranging from 20% to 1% (‘Long_COVID’); and the legal rules, including whether vaccine passports were required (needed, not needed; ‘Green_pass’) and whether vaccine mandates existed (‘Vaccine_mandate’). We tested no vaccine mandate versus a mandate for all adults with a high fine of 1,500 Euros and a more limited mandate for people aged 50+ with a lower fine of 100 Euros. We calculated AMCEs. Data are presented as AMCE estimates ± 95% CIs. Exact *P* values are shown in Supplementary Files 9–12. In total, 6,357 respondents participated in this experiment (Austria: *n* = 3,187; Italy: *n* = 3,170).

In experiment 2, we investigated the role of the wider information environment. We showed each respondent two fictional media reports and asked them to assess based on which report they would trust the vaccine more (binary choice) and rate their likelihood to get vaccinated for each scenario on a 0–10 scale (ratings). The manipulated attributes of each media report (Fig. 2) included the consensus of experts, celebrity endorsement, the prevalence of Long COVID and the legal rules, such as vaccine passports and vaccine mandates. For the full wording of the experimental treatments and outcome variables, see the Methods section and Supplementary File 4. The results are shown in Fig. 2.

Our results confirm that communicating expert consensus will likely increase COVID-19 vaccinations^31^. Specifically, the triple vaccinated showed a lower likelihood to get vaccinated when scientists (−0.133, CI −0.263 to −0.002) or physicians (−0.161, CI −0.293 to −0.030) disagreed in the dissensus scenarios as compared to when there was a consensus. Trust in the vaccine was significantly lower in both countries when people were facing expert dissensus. Also, a lack of expert consensus negatively affected trust in vaccinations among those respondents who had three or more vaccinations.

Likewise, celebrity endorsement was most relevant for the triple vaccinated. They showed an increased likelihood to get vaccinated if a celebrity recommended getting vaccinated (0.169, CI 0.039–0.299) or fell ill and regretted not having been vaccinated (0.134, CI 0.004–0.264) as compared to when a celebrity was openly opposed to vaccinations. Although the lower bounds of the CIs are close to zero for the vaccination intentions, celebrity endorsement and regrets about not getting vaccinated sooner also contributed to significantly higher levels of trust in vaccinations in both countries. Only the unvaccinated group showed slight signs of backlash, placing less trust in vaccinations when endorsed by a celebrity.

The likelihood to get Long COVID after the infection and vaccine passports mattered only in a few instances. For example, in Austria, we saw a tendency for heightened readiness to get vaccinated at a 20% likelihood to get Long COVID (0.213, CI 0.030–0.395). A high likelihood to get Long COVID (0.036, CI 0.014–0.058) and the requirement of a vaccine passport (0.040, CI 0.024–0.056) also contributed significantly to greater trust in the vaccine among the triple vaccinated. Finally, media reports about a vaccine mandate showed no visible effects on the likelihood to get vaccinated but strongly affected the trust in vaccines negatively. The most substantial negative effect on trust in vaccinations was observed for a general vaccine mandate applying to the adult population with a high fine (1,500 Euros), but a more limited mandate for adults aged 50+ with a lower fine (100 Euros) also undermined trust in vaccinations.

## Discussion

In addition to the challenge of vaccine hesitancy, vaccine fatigue is becoming a growing concern for public health officials due to waning immunity and the emergence of new virus variants that make repeated booster vaccinations necessary^14,47^. Indeed, the uptake of boosters has so far remained below expectations in many countries^2,3^. The definition of concepts such as vaccine hesitancy and vaccine fatigue has not always been clear in the literature^4^. Here we refer to both of these concepts in a broad sense as an umbrella term, describing a low or intermediate propensity to get vaccinated either for the first time (vaccine hesitancy) or repeatedly (vaccine fatigue), covering a broad spectrum, including those in a state of indecision and uncertainty as well as those who oppose and refuse vaccination or repeated vaccination^48,49^, but we acknowledge that, also, more narrow conceptions coexist. Although vaccine hesitancy already has a vast literature, including some studies on COVID-19 vaccine hesitancy, less is known about vaccine fatigue, specifically in the context of COVID-19 (ref. ^8^), which motivated this study to explore its determinants. Our results provide evidence on how to encourage vaccine uptake in the face of growing levels of vaccine fatigue. Under partly unknown future circumstances, we simultaneously evaluated the role of multiple contextual features and possible interventions by presenting to study participants various hypothetical scenarios in two conjoint experiments in two countries, Austria and Italy. The results revealed some overarching general patterns but also showed some nuanced and complex findings that need to be discussed and looked at as a whole to deduce actionable advice.

The first question assessed in this study was whether future vaccination campaigns should rather be conceptualized more as a one-size-fits-all (uniform approach) or whether group-specific characteristics should be taken into account (tailored approach). Our results suggest distinguishing campaigns between primary and booster vaccinations. Although this is in line with research done in earlier stages of the pandemic^39^, our results also showed additional variation between those who have not had the first booster and the triple vaccinated, suggesting that further group differences may need to be considered when designing vaccination campaigns. To a lesser extent, we also observed between-country differences—for example, regarding the relevance of information on Long COVID, which mattered more in Austria than in Italy. All in all, these patterns underline our first piece of actionable advice that instruments of vaccination campaigns need to be tailored and tested before the campaign rollout, taking into account characteristics of the national context and the different target groups based on their vaccination status.

The second question investigated was about how different groups can and should be addressed by vaccination campaigns. Although it seems warranted from a medical perspective to emphasize the need to ‘close the vaccination gap’ and to focus efforts mainly on the unvaccinated, closing this gap may be difficult to achieve, as our results showed that the unvaccinated score low on trust in institutions and are the least likely to get vaccinated across all scenarios. Given that both Austria and Italy had temporarily considered some type of vaccine mandate during the pandemic, it seems that most of those who can be reached by vaccination campaigns have already been vaccinated at least once, with only a few exceptions^6^. Only campaign messages conveying a sense of community and emphasizing the need to hold together to overcome the crisis were effective in promoting behavioral change in the unvaccinated group. Otherwise, we mainly found signs of reactance within this group, particularly in response to legal rules (for example, the requirement of a vaccine passport or a vaccine mandate), which were found to undermine trust in the vaccine. Taken together, these results suggest that the most socially agreeable way to encourage primary vaccinations would be to focus on promoting community spirit instead of relying on stricter policy interventions. In the medium term and long term, it would also be essential to address underlying factors contributing to vaccine hesitancy, such as low trust in institutions, as the overabundance of (often controversial) information, rumors and conspiracy theories^50^ are likely to undermine public confidence in vaccines and contribute not only to vaccine hesitancy but also to growing vaccine fatigue.

Those who have been vaccinated once or twice were strongly encouraged by positive incentives, both in the form of cash or vouchers and the availability of new vaccines, including both variant-adapted and non-mRNA vaccines. Although the latter result was only marginally statistically significant, it may hint at the need to gain a better understanding of heterologous vaccinations and to obtain regulatory approval for new non-mRNA vaccines as boosters. We found no significant communication effects for this group with regard to campaign messages, expert consensus, the role of celebrities or information on Long COVID. Sending out written messages might not be as effective as in the influenza context^21^ to increase vaccine uptake in this particular group. One reason for this could be the high levels of fatigue and growing tendency to actively avoid information on the COVID-19 pandemic more generally^51^. This might be a sign that communicative mobilization cannot be sustained over the long term and that it might be necessary to implement a more institutionalized form of procurement for vaccines that require regular updates in conjunction with a scheme of positive incentives to counteract vaccine fatigue.

Finally, offering additional incentives and strategizing about vaccination campaigns for the triple vaccinated may appear like a superfluous exercise—one may expect them to go and get the necessary booster(s) anyway and under any circumstances. Our results, however, suggest otherwise: even implementing seemingly trivial costs (20 Euros) to vaccines can strongly deter the triple vaccinated from translating their positive attitudes into actual behavior. We found a high degree of cost sensitivity among the triple vaccinated. This was the strongest effect across both conjoint experiments and most consistent across the two countries. Cost-free and easy access to vaccines as well as creating awareness of when and how to get the vaccine, therefore, are likely to remain the mainstay for any vaccination campaign to succeed. The experiments also revealed several other features that could further reinforce vaccination readiness and slow vaccine fatigue among the triple vaccinated: adapted vaccines, medical consensus and celebrity endorsement. Furthermore, the results showed that campaign messages emphasizing protecting oneself against severe disease, protecting vulnerable peers, protecting the healthcare system, community spirit and self-efficacy resonated well in this group. Considering that the triple vaccinated were the largest group in both countries, failing to (re)activate and (re)mobilize this group is likely to result in booster vaccination rates falling far short of expectations.

A strength of the conjoint design of this study is that it allowed us to compare the relevance of multiple contextual features and possible interventions in future scenarios all at once. This is particularly relevant in a situation like the current one, where future circumstances, such as the possible emergence of new virus variants, are partly unknown, which can create many contingencies for policymakers. Another strength is the sample size that facilitated conducting subgroup comparisons between groups based on their immunization status and between two countries. We advance the existing knowledge by shedding light on how subgroups of the population would need to be addressed to increase the vaccination rate in the current situation characterized by growing vaccine fatigue. We also show which instruments that had previously been found to be effective may no longer work and may even undermine trust in the vaccines.

Considering the limitations, one aspect concerns the generalizability of the findings. We acknowledge that other countries have decided to offer second boosters mainly to those most at risk, particularly older adults and those with pre-existing conditions of any age. Despite this, we think that the results are, to some extent, informative and applicable to those countries with somewhat differing recommendations for two reasons. First, the study population of triple-vaccinated individuals were, on average, from older age cohorts, similar to those for whom a second booster is recommended in other countries. Second, the results for those who have not yet received primary vaccinations or a first booster are not affected by differing recommendations regarding second boosters, and many countries generally recommend three vaccinations since the emergence of the Omicron variant. Overall, the number of previous vaccinations is one of the strongest predictors of future vaccination intentions, as it captures much of the underlying psychology (Supplementary File 10) and is a stronger effect modifier than the national context in this study. Therefore, we think that the findings regarding the conditional effects for the subgroups by vaccination status should vary only rather modestly across different national settings.

Another potential concern is the multiplicity of testing, as the conjoint design produces a high number of coefficients due to its exploratory nature. To evaluate the relevance of this concern, we adjusted the significance levels and report them in the Appendix (Supplementary Files 11–18). However, the additional degree of uncertainty arising from the multiplicity of testing affects only those estimates where the lower bounds of the 95% CIs were already quite close to zero. As we already took into account the greater uncertainty of these estimates in our interpretation, our substantive conclusion overall remains unchanged. In this context, in terms of the substantive interpretation, the magnitude of effects (point estimates) is considered to be more relevant than their precision (*P* values), and the gradual nature of precision estimates should always be taken into account^52,53^.

Finally, possible limitations regarding external validity are to be considered. Although conjoint experiments enable possible impacts of future scenarios to be explored, reading a hypothetical scenario description may differ in considerable ways from experiencing how such a situation plays out in real life. The exact effect size is somewhat contingent on how tangible the conditions become for individuals. For example, our results suggest that it could be difficult to convince the unvaccinated and the partially vaccinated of the need for (further) vaccination in the event of the emergence of a more severe virus variant. We, therefore, think that vaccine fatigue could be a considerable obstacle in such a situation, potentially leading to detrimental outcomes. However, whether it would actually come to that would also depend on how markedly the events in such a scenario would happen in reality. To address the limitation of external validity, the effectiveness of some suggested measures to increase adherence to repeatedly needed vaccinations, such as bonus programs among relevant target populations, could be evaluated by future research in randomized controlled trials, as they have been conducted in the context of influenza vaccination^54,55^ or other programs encouraging healthy behavior change^56^. Future research in this regard could address COVID-19 and influenza vaccine fatigue jointly and evaluate the effectiveness of such a coordinated effort in future investigations.

Overall, the results enhance understanding of vaccine acceptance in the context of growing vaccine fatigue. Several actionable points can be deduced from the analysis: (1) test the design and instruments of vaccination campaigns with target groups; (2) keep the cost-free provision of vaccines and easy access to vaccination sites in which even seemingly trivial costs could be strongly discouraging; (3) promote community spirit and set measures to strengthen social cohesion and institutional trust in the long term; (4) offer and communicate about new and adapted vaccines to encourage booster uptake; (5) consider moving from communicative mobilization to more institutionalized bonus programs with positive incentives for booster vaccinations in the long term, if budgetary constraints allow; (6) carefully assess the risks and benefits of stricter policy instruments involving legal requirements, such as vaccine passports and vaccine mandates, which bear a risk of backlash; and (7) facilitate consensus-building among medical professionals and scientists by supporting research and making relevant evidence readily available.

Considering the group sizes and the size of treatment effects, vaccination campaigns should not neglect to mobilize the triple vaccinated, as even minimal costs could deter them from translating their fairly positive attitudes toward vaccination into action. Doing so would result in low booster uptake remaining considerably below expectations, which might put the most vulnerable at risk due to waning immunity and the possible emergence of new virus variants. We hope that these results will inform policymakers, health professionals and experts in charge of conducting COVID-19 vaccination campaigns and responsible for informing the public. Future campaigns should consider the recommendations outlined in this study while also incorporating appropriate evaluations to measure their success and gain further insights.

## Methods

### Survey design and conjoint experiments

The survey was planned and designed in early summer 2022, with the goal of providing scientific evidence for potential vaccination campaigns in future scenarios of the pandemic. Conjoint experiments were used previously to analyze how a broad range of factors affects vaccine acceptance, and the research design allows exploring the implications of various future scenarios. We reviewed the literature on vaccination readiness to identify relevant attributes to be manipulated in the experiment^8,24–39^. We also consulted with practitioners in the public health sector and country experts to assess what kind of new and current developments might have consequences for vaccine uptake in future scenarios. We found that both the objective conditions, such as virus variants and availability of new vaccines, as well as more subjective factors, such as motivations, were likely to play a role. Also, the evidence suggested that, in addition to the vaccination campaigns, media coverage on vaccinations and the wider information environment were likely to affect the public mood when further rounds of vaccinations would become necessary. We, therefore, included two experiments to cover both of these aspects. In both experiments, we varied attribute levels randomly to assess which components of a multidimensional treatment would be influential. The complete list of attributes and their levels as well as the wording of the prompt and follow-up questions are available in Supplementary Files 3 and 4. Note that study participants evaluated only hypothetical scenario descriptions and were not assigned any medical treatment or product.

Besides the conjoint experiments, the survey included questions on sociodemographics, general health, attitudes toward vaccination, trust in institutions and emotions, such as the experienced levels of pandemic fatigue. The standardized questionnaire was designed for interviews to last for about 15 minutes. All questions were asked in a closed-ended format. We used nominal scales, five-point Likert-type scales as well as numerical rating scales (for example, 0–10 rating scale) to record the responses. The translations of the questions were done by native speakers and checked by experts familiar with the local conditions and vaccination discourse in Austria and Italy.

The questionnaire was then programmed and tested. Before the questionnaire was fielded, the survey was pilot tested by the research team and laypersons, and some minor adjustments were made based on the feedback. The survey was then fielded by the survey company, and the interviews were realized as computer-assisted web interviews (CAWIs).

### Details on experiment 1—a hypothetical vaccination campaign

Experiment 1 was designed to assess scenarios for a hypothetical vaccination campaign. The discussion with experts in public health revealed that there was uncertainty about the evolution of virus variants, and we included this attribute to evaluate to what extent the severity of virus variants could influence vaccination decisions. The review of the literature revealed that properties of available vaccines, costs/incentives and campaign messages could affect vaccine uptake. Discussions with country experts revealed that some people seemed to be waiting for new vaccines to become available, with some waiting for inactivated virus vaccines and some waiting for vaccines adapted to the Omicron variants. Regarding campaign messages, the literature suggested that emotions may play a role in vaccine uptake. To convey emotions, we used a testimonial by a fictive person (randomizing age group and gender) making a statement about his or her motivation to get vaccinated. We included statements about economic and health risks at the individual, interpersonal and collective levels. Taking up advice from the expert consultations, we also included messages emphasizing a sense of community and self-efficacy. The wording of all attributes and levels is listed in Supplementary File 3 (see Supplementary Table 3.1: English translation; Supplementary Table 3.2: original version in German; and Supplementary Table 3.3: original version in Italian).

### Details on experiment 2—media communication on vaccinations

Experiment 2 was designed to assess scenarios of hypothetical media coverage on vaccinations. Recent research has demonstrated the importance of communicating expert consensus to increase vaccinations. However, media coverage often tries to balance perspectives, even when there is far-reaching consensus (‘false balance’). We, therefore, included fictional reports about a TV discussion round as an attribute in the experiment. Previous research also suggested that celebrity endorsement might play a role. As recent coverage on vaccination included both celebrities endorsing vaccinations but also opposition to vaccination by celebrities, we included a brief fictional report on celebrity behavior. The discussion with public health experts further revealed that there was great uncertainty about the risks of Long COVID. Hence, we included an attribute varying the likelihood change of getting Long COVID after infection. Finally, previous research has shown that legal rules, such as vaccine passports and vaccine mandates, may matter for vaccinations. To evaluate whether such instruments would be suitable for increasing vaccinations, we included an attribute for the requirement of a vaccine passport and two kinds of vaccine mandates. The wording of all attributes and levels is listed in Supplementary File 4 (see Supplementary Table 4.1: English translation; Supplementary Table 4.2: original version in German; and Supplementary Table 4.3: original version in Italian).

### Participants

We used population-representative quotas to recruit the respondents from the commercial online access panel by Marketagent GmbH (certified under International Organization for Standardization 20252), encompassing more than 135,000 registered panelists in Austria and 87,000 in Italy. The target population in each country was residents aged 14+ years. Note that, from the age of 14, adolescents in Austria and Italy can decide for themselves, without their parents’ consent, whether they want to be vaccinated or not. The survey was initially opened for 61,503 Austrians and 73,077 Italians and closed after the target quotas were reached in each country. Eventually, 3,187 participants from Austria and 3,170 participants from Italy took part in the study after providing informed consent. The participation rate was overall similar to comparable studies^8,57–59^. The quota targets and actual values in the sample are shown in Supplementary File 1. Note that, although we aimed to make the sample structure match as closely as possible to the targets, some subgroups of the population remain hard to reach for online surveys (for example, very high age and language minorities).

### Information about the countries

During several stages of the global vaccine rollout, the German-speaking countries showed the lowest COVID-19 vaccination rates in Western Europe, with Austria having long held the highest share of unvaccinated people among these countries^60–62^. In August 2022, Austria stood at a rate of 77.1% of people who completed the primary course, and 59.2% received the first booster^41^. Flattening vaccination rates, however, show hesitant booster uptake. Austria was, therefore, among the first countries that announced a general COVID-19 vaccination mandate, which it has recently withdrawn after experiencing increasing societal polarization over this issue^63^. Although, like Austria, Italy was also struggling with booster campaigns at the beginning of autumn 2022, it was ranked better in vaccination uptake among the European countries. In August 2022, 80.2% of Italians were first-course uptakers, and 71.5% received boosters^11,42^. Italy also first introduced and later withdrew a vaccination mandate, similar to Austria, but with the difference that it was only for healthcare workers^64^. Vaccine fatigue, however, is also high in many other countries. For instance, it has been reported that booster uptake in Canada was stalling^2^, and, according to recent news reports, this is similar in the United States^3^.

### Sample size calculation

We calculated a minimum sample size of 2,017 for a conjoint experiment based on the size of the population of Austria/Italy, desired confidence levels of 95% and error margins of 2%. We also took into consideration that it might not be possible to motivate some groups in the population for further vaccinations due to vaccine fatigue. Although, in July 2022, about 77% of Austrians and 85% of Italians had received at least one dose, only 59% of Austrians and 72% of Italians had received the first booster vaccinations. Based on these numbers, we estimated that only two-thirds of the respondents in our sample might be susceptible to treatment, whereas about one-third would refuse vaccination under any scenario. We, therefore, opted for a sample size of approximately 3,000 respondents for each country.

### Statistical methods

**Extended Data Fig. 3:**
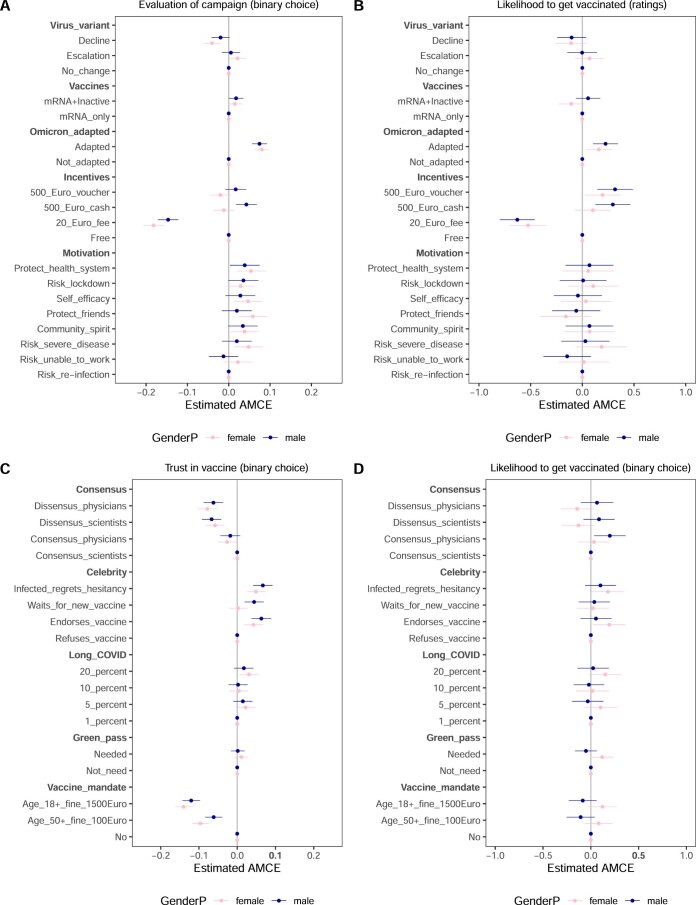
Results of Experiment 1 (A,B) and Experiment 2 (C, D) stratified by gender. This figure presents the disaggregated results of the two conjoint experiments stratified by gender. Gender was assessed by respondents’ self-reports in the survey. Therefore, we used the term "gender" throughout this work because of the self-reported nature of the data. We included only male and female gender as categories in this analysis since the number of participants who indicated ‘non-binary/other’ gender was too small to obtain reliable results for this group (depicted in Supplemental File 1). Panels A and B show the results of Experiment 1 by gender. Panel A refers to the binary measure of campaign evaluations, whereas Panel B depicts the ratings of the likelihood of getting vaccinated. Overall, there are only small differences between gender groups, and confidence intervals mostly overlap. One notable difference is that men evaluated vaccination campaigns with cash incentives as more appealing than women. Also, regarding the likelihood of getting vaccinated, men seemed to respond somewhat more strongly to positive incentives, although the confidence intervals between the gender groups overlap in this case. Panels C and D show the results of Experiment 2 by gender. Again, we find only small differences across gender groups. In fact, confidence intervals overlap for all of the treatment conditions. ‘GenderP’ means the gender of the survey participants as opposed to the gender of the testimonial in the case vignettes. We calculated Average Marginal Component Effects (AMCEs). Data are presented as AMCE estimates +/−95% confidence intervals. Exact p-values are shown in Supplemental Files 13–16. In total, 6,357 respondents participated in both experiments (Austria: n = 3,187; Italy: n = 3,170). For further information on the number of male and female respondents included in the sample, see Supplemental File 1. We also report the gender composition of the vaccination status groups in Supplemental File 2.

We used descriptive statistics for sociodemographic variables and attitudes to characterize the sample. We present results in tabular form and as box or bar plots as appropriate and included them in Extended Data Figs. 1 and 2. To evaluate the impact of the attribute levels in the conjoint experiments, we computed average marginal component effects (AMCEs) with 95% CIs for the binary responses. We display the AMCEs by country and vaccination status as coefficient plots for ease of interpretation. We show the results with and without correction of multiplicity. For the full estimation tables, see Supplementary Files 5–12. We included a disaggregated description of the results by gender in Extended Data Fig. 3 and Supplementary Files 13–16. For reporting, we adhered to the Strengthening the Reporting of Observational Studies in Epidemiology (STROBE) checklist (Supplemental File 17).

### Assessment of data quality

To assess data quality, we investigated non-response patterns. Specifically, we calculated the number of times respondents did not give substantial answers (‘Don’t know’, ‘No answer’). We flagged an interview as suspicious when respondents gave an unsubstantial answer more than 50% of the time. We found no Austrian particpants and 151 Italian participants for whom this was true and performed the analyses with and without these participants. There were no changes in the results of the conjoint experiments, and, for this reason, we did not exclude these participants from the analyses.

### Details on how we dealt with missing values

Respondents were required to answer the questions on the conjoint experiments to complete the interview. For the binary choice questions, respondents were instructed to spontaneously choose one of the two options if they felt indifferent. Therefore, there are no missing values on our outcome variable. The measures that were included in the survey for descriptive purposes, such as sociodemographics, institutional trust and emotions, offered opt-out options (‘Don’t know’, ‘No answer’). When calculating descriptive statistics, such responses were excluded from the analysis.

### Study registration

The conjoint experiments were not pre-registered, which was not required under local guidelines and did not seem appropriate, given the exploratory nature of the research question. A conjoint experiment is a survey-based, exploratory research method that attempts to understand how people make complex choices. It follows a cross-sectional, observational design and collects data only once. It includes hypothetical scenarios, which we developed for the present study in a way that all attribute levels were realistic and all possible combinations were plausible in both country contexts. Attribute levels were randomized, and not all participants saw the same levels. The experiment, thus, provides initial, exploratory evidence through which channels and in which way we could reach which target groups. Based on the findings from a conjoint experiment like ours, one can develop a targeted intervention and then test it in a subsequent clinical trial. Similar studies were also not registered as clinical trials^24–26^.

### Reliability and validity of survey instruments

This study used several survey instruments to characterize the study population (Supplementary Files 4–6) and two survey experiments to assess the effect of various contextual features and interventions (Supplementary Files 7 and 8) that can influence the likelihood to get vaccinated. Specifically, to characterize the underlying psychology of different subgroups in the sample, we used measures to capture (1) emotions, (2) trust in various institutions and (3) attitudes toward COVID-19 vaccination. In the experiments, we manipulated the virus variant, the availability of vaccines, costs/incentives, campaign messages, consensus of experts, celebrity endorsement, information on Long COVID and legal rules, such as vaccine passports and vaccine mandates. We measured their impact on the evaluation of the campaign (experiment 1), trust in the vaccine (experiment 2) and the likelihood to get vaccinated (both experiments). In the following, we discuss aspects related to the reliability and validity of the survey measures used in this study.

Regarding the measurement of emotions, we adopted the approach to assess a set of distinct emotions. The reliability and validity of the measurement of emotions in surveys have been evaluated, for instance, by Marcus et al.^65^. In their study, they asked respondents to describe how they felt using a set of distinct emotions (for example, being worried, hopeful, angry, etc.). They showed that both radio buttons and continuous slider scales are highly reliable instruments for capturing emotions in surveys. According to their results, a more fine-grained response scale with more than five categories was preferable to allow for more nuanced responses and better discrimination, which was why we used an 11-point scale to record the responses. They also assessed the construct validity of the measurement of emotions. Among other things, they assessed to what extent emotions correlated in expected ways with the interest in novel information, confirming, for example, that anxiety is positively related to information seeking. We built on this approach and added feeling ‘tired’ or ‘exhausted’ as items to the list of emotions, as Lilleholt et al.^66^ have shown that the feeling of fatigue is negatively related to information seeking, as well as preventive behavior, such as physical distancing, wearing masks and hygiene. In our data, avoiding information seeking was correlated with feeling tired (*r* = 0.3, *P* < 0.001).

To measure trust in institutions, we used an item battery with a list of institutions that respondents evaluated on an 11-point scale. Due to their relevance as possible sources and multipliers of health-related information in the context of the pandemic, we included pharmaceutical companies, the healthcare system, schools and science, in addition to the government, parliament and the media. The theoretical underpinnings for the measurement go back to political culture studies and studies of political system support^67,68^. Similar measures have been conventionally used in many surveys, such as the European Social Survey (ESS), the World Value Survey (WVS), the European Union Statistics on Income and Living Conditions (EU-SILC), the European Quality of Life Survey (EQLS) and the Eurobarometer or the Gallup World Poll (GWP). The reliability of such measures has been evaluated, for instance, in an Organization for Economic Cooperation and Development (OECD) working paper by González and Smith^69^. They reported correlations that are in line with a high degree of reliability of the measurement and found a good performance in terms of construct validity.

Various models of the psychological antecedents of vaccination have been discussed in the literature (see, for example, Betsch et al.^70^ for an overview), and this literature has identified many psychological factors affecting vaccine acceptance. Based on this research, as well as based on insights from qualitative interviews conducted by colleagues from the ‘Solidarity in times of a pandemic’ (SOLPAN) project^71^, a new item battery to capture attitudes toward COVID-19 vaccination was developed in the context of the Austrian Corona Panel Project^57^ at the beginning of the initial rollout (for the results from the item battery, see Extended Data Fig. 2). These items were found to be strongly correlated with the number of vaccinations, jointly explaining more than 50% of the variance of the COVID-19 vaccination status. As can be seen in Supplementary File 6, these items also showed the expected relationships in our sample. In the context of this study, we also added two additional items to capture perceptions that (1) the COVID-19 vaccines provide only limited protection against infection (‘Vaccination does not help, you still get ill’) and (2) the emergence of the new virus variants reduce the need for vaccination (‘The current virus variant is mild, so I don’t need vaccination’). Also, these items showed the expected relationships with vaccination status. In our sample, the items explained 61% of the variance of vaccination readiness. As attitudes and vaccination status mapped quite closely to each other, and as this seemed most relevant in practical terms for vaccination campaigns, we conditioned on the vaccination status in our analysis. An analysis using psychological factors as moderators did not yield fundamentally different insights.

Finally, experimental treatments and response scales were similar to those used by previous research on interventions for increasing COVID-19 uptake (for a review, see Batteux et al.^22^). Thus, similar measures have been conventionally used and can be considered as established instruments in the relevant literature (see also the cited literature on properties of vaccines^24–26^, communication (for example, campaign messages^27–30^, expert consensus^31^ and celebrity endorsement^32–34^), costs/incentives^8,34–36^ and legal rules (for example, vaccine passports^36,37^ and vaccine mandates^38,39^)). In terms of predictive validity of such survey measures for actual behavior, note that reported vaccination intentions from panel surveys were highly predictive of actual vaccination behavior in the context of the COVID-19 pandemic^72^. Thus, considering all aspects related to the measurement, we think that the survey instruments and experimental designs used provide a reliable and valid measurement.

### Ethics statement

All survey participants provided informed consent to the survey company that carried out the fieldwork. Only anonymized data were received and analyzed. Research ethics approval for this study was not required according to institutional and national guidelines, such as the Medical Devices Act of Austria.

### Reporting summary

Further information on research design is available in the Nature Portfolio Reporting Summary linked to this article.

## Online content

Any methods, additional references, Nature Portfolio reporting summaries, source data, extended data, supplementary information, acknowledgements, peer review information; details of author contributions and competing interests; and statements of data and code availability are available at 10.1038/s41591-023-02282-y.

## Supplementary information

Supplementary Information

Reporting Summary

## Data Availability

The raw data generated in this study are publicly available at 10.7910/DVN/3R2CMT (Harvard Dataverse).

## Code availability

We published the analytic code associated with this study at 10.7910/DVN/3R2CMT (Harvard Dataverse).
