# Supplementary material for: Determinants of COVID-19 vaccine fatigue
Source: Nat Med. 2023 Mar 27;29(5):1164–71. doi: 10.1038/s41591-023-02282-y (PMC10202806; doi:10.1038/s41591-023-02282-y)
Supplement: Supplementary file 1 — Supplementary Information [file 41591_2023_2282_MOESM1_ESM.pdf]

nature medicine

Article

https://doi.org/10.1038/s41591-023-02282-y

Determinants of COVID-19 vaccine fatigue

In the format provided by the authors and unedited

Appendix with Supplemental Information

Table of Contents

SUPPLEMENTAL FILE 1. SAMPLE CHARACTERISTICS 2
SUPPLEMENTAL FILE 2. SOCIODEMOGRAPHIC CHARACTERISTICS OF VACCINATION STATUS GROUPS 3
SUPPLEMENTAL FILE 3. DETAILS ON EXPERIMENT 1 – A HYPOTHETICAL VACCINATION CAMPAIGN 4
SUPPLEMENTAL FILE 4. DETAILS ON EXPERIMENT 2 – MEDIA COMMUNICATION ON VACCINATIONS 9
SUPPLEMENTAL FILE 5. EFFECTS ON THE EVALUATION OF THE CAMPAIGN BY COUNTRY (EXPERIMENT 1)........ 13
SUPPLEMENTAL FILE 6. EFFECTS ON THE LIKELIHOOD TO GET VACCINATED BY COUNTRY (EXPERIMENT 1)........ 14
SUPPLEMENTAL FILE 7. EFFECTS ON THE EVALUATION OF THE CAMPAIGN BY VACCINATION STATUS (EXPERIMENT 1) 15
SUPPLEMENTAL FILE 8. EFFECTS ON THE LIKELIHOOD TO GET VACCINATED BY VACCINATION STATUS (EXPERIMENT 1) 17
SUPPLEMENTAL FILE 9. EFFECTS ON TRUST IN THE VACCINE BY COUNTRY (EXPERIMENT 2)........ 19
SUPPLEMENTAL FILE 10. EFFECTS ON THE LIKELIHOOD TO GET VACCINATED BY COUNTRY (EXPERIMENT 2)........ 20
SUPPLEMENTAL FILE 11. EFFECTS ON TRUST IN THE VACCINE BY VACCINATION STATUS (EXPERIMENT 2)........ 21
SUPPLEMENTAL FILE 12. EFFECTS ON THE LIKELIHOOD TO GET VACCINATED BY VACCINATION STATUS (EXPERIMENT 2) 23
SUPPLEMENTAL FILE 13. EFFECTS ON THE EVALUATION OF THE CAMPAIGN BY GENDER (EXPERIMENT 1) 25
SUPPLEMENTAL FILE 14. EFFECTS ON THE LIKELIHOOD TO GET VACCINATED BY GENDER (EXPERIMENT 1) 26
SUPPLEMENTAL FILE 15. EFFECTS ON TRUST IN THE VACCINE BY GENDER (EXPERIMENT 2)........ 27
SUPPLEMENTAL FILE 16. EFFECTS ON THE LIKELIHOOD TO GET VACCINATED BY GENDER (EXPERIMENT 2) 28
SUPPLEMENTAL FILE 17. STROBE STATEMENT........ 29

Supplemental File 1. Sample characteristics

	Italy (N=3,170)		Austria (N=3,187)	
	Target	Sample	Target	Sample
Gender				
Male	50%	1,467 (46%)	49.8%	1,542 (48.3%)
Female	50%	1,532 (48%)	50.1%	1,629 (51.0%)
Non-binary/Other	0%	171 (5.0%)	0%	16 (1.0%)
Age groups				
14 to 24 years	14.0%	440 (13.9%)	15.0%	491 (15.4%)
25 to 34 years	13.7%	439 (13.8%)	17.5%	561 (17.6%)
35 to 44 years	16.0%	533 (16.8%)	17.0%	509 (16.0%)
45 to 54 years	20.6%	651 (20.5%)	19.3%	606 (19.0%)
55 to 64 years	19.2%	623 (19.7%)	18.1%	595 (18.7%)
65+ years	16.6%	484 (15.3%)	13.2%	425 (13.3%)
Education				
Low/Medium	40.3%	1,182 (37.3%)	67.9%	2,187 (68.6%)
High	59.7%	1,973 (62.2%)	32.1%	970 (30.4%)
Other	0%	15 (0.5%)	0%	30 (0.9%)
Regions: Italy				
Piemonte	7.2%	258 (8.1%)		
Valle d'Aosta	0.2%	8 (0.3%)		
Trentino Alto	1.8%	44 (1.4%)		
Adige Veneto	8.2%	210 (6.6%)		
Friuli Venezia Giulia	2.0%	72 (2.3%)		
Giulia Liguria	2.6%	85 (2.7%)		
Lombardia	16.9%	548 (17.3%)		
Toscana	6.2%	163 (5.1%)		
Umbria	1.5%	37 (1.2%)		
Lazio	9.7%	313 (9.9%)		
Abruzzo	2.2%	57 (1.8%)		
Emilia-Romagna	7.5%	237 (7.5%)		
Sardegna	2.7%	109 (3.4%)		
Marche	2.5%	74 (2.3%)		
Campania	9.5%	313 (9.9%)		
Molise	0.5%	20 (0.6%)		
Puglia	6.6%	235 (7.4%)		
Basilicata	0.9%	29 (0.9%)		
Calabria	3.1%	89 (2.8%)		
Sicilia	8.1%	269 (8.5%)		
Regions: Austria				
Vorarlberg			4.4%	132 (4.1%)
Tyrol			8.5%	264 (8.3%)
Salzburg			6.3%	210 (6.6%)
Styria			14.2%	460 (14.4%)
Carinthia			6.4%	206 (6.5%)
Upper Austria			16.6%	527 (16.5%)
Lower Austria			18.9%	593 (18.6%)
Vienna			21.4%	669 (21.0%)
Burgenland			3.4%	126 (4.0%)

Note: Comparison of quota targets used for recruitment of participants and actual values in the sample.

Supplemental File 2. Sociodemographic characteristics of vaccination status groups

Vaccination status	N (%)	Vaccination readiness (mean±SD)	Gender (% male)	Age (mean±SD)	Education (% highest third)
Not	1618 (25%)	3.4±3.0	**50%**	44±17	13%
1 or 2	878 (14%)	4.7±2.6	48%	38±16	16%
≥3	**3861 (61%)**	**6.6±2.5**	48%	**47±16**	**22%**

Notes: Highest value per column in bold. Vaccination Readiness is the average of all ratings across all scenarios across both experiments for the likelihood to get vaccinated (measured on a 0-to-10 scale). Gender information is without "non-binary"/"other".

Supplemental File 3. Details on experiment 1 – A hypothetical vaccination campaign

The introductory sentence for the experiment read as follows: “Below, we present several hypothetical scenarios for calls for further COVID-19 vaccinations. The scenarios include fictional calls for COVID-19 vaccination.” Respondents were then shown two scenarios at a time and were asked to answer two follow-up questions (Q1, Q2). Levels were randomly assigned with equal probabilities and repeatedly recombined, with all combinations being plausible and realistic. The procedure was repeated twice so that each respondent had to evaluate four scenarios in total.

**Scenario 1**	**Scenario 2**
Virus variants are emerging, *<Variant, 1 of 3>* and infection rates are increasing. There is a general vaccination recommendation for everyone 14 years and older.	Virus variants are emerging, *<Variant, 1 of 3>* and infection rates are increasing. There is a general vaccination recommendation for everyone 14 years and older.
Available vaccines:<br>*<Vacc, 1 of 2>*	Available vaccines:<br>*<Vacc, 1 of 2>*
Versions of the vaccines adapted to Omicron are <Omic*, 1 of 2>.*	Versions of the vaccines adapted to Omicron are <Omic*, 1 of 2>.*
<Incen, 1 of 4>	*<Incen, 1 of 4>*
<Name, age>:<br>*"<Motiv, 1 of 8>.*<br>That's why I'm going to get vaccinated.<br>And what's your plan for the fall?"	*<Name, age>:*<br>*"<Motiv, 1 of 8>.*<br>That's why I'm going to get vaccinated.<br>And what's your plan for the fall?"

Q1. Please imagine the following hypothetical situations for the fall and read the vaccination calls carefully.

In which of these two scenarios does the vaccination call appeal to you more? If you find neither or both calls equally appealing, please decide spontaneously. (Binary choice)

- Scenario 1
- Scenario 2

Q2. On a scale of 0 to 10, how likely is it that you would heed these calls for vaccination and get vaccinated against COVID-19? (Rating scale)

- Scenario 1: 0 = “would definitely not get vaccinated” to 10 “would definitely get vaccinated”
- Scenario 2: 0 =” would definitely not get vaccinated” to 10 “would definitely get vaccinated”

<u>Table S3.1: English version</u>

**Attributes**	**Levels**
Virus variants (\`Variant\`)<br>• *Decline*<br>• *No change*<br>• *Escalation*	• which are <u>less dangerous </u>than the current virus variant.<br>• <u>similar to </u>the current virus variant.<br>• <u>more dangerous </u>than the current virus variant.
Type of vaccine (\`Vacc‘)<br>• *mRNA vaccines only*<br>• *mRNA vaccines plus inactivated vaccines*	• mRNA vaccines: BioNtech-Pfizer, Moderna<br>• mRNA vaccines: BioNtech-Pfizer, Moderna

Attributes	Levels
	Inactivated vaccines: Novavax[1], Valneva
Adaptation to Omikron (`Omic‘)	● available<br>● not available
Incentives (`Incen‘)<br>● *free of charge*<br>● *not free of charge*<br>● *Premium in kind*<br>● *Cash reward*	● The vaccination is free of charge.<br>● The vaccination costs 20 Euros.<br>● For the vaccination, you will receive a shopping voucher for 500 Euros as an expense allowance.<br>● For the vaccination you will receive 500 Euros as an expense allowance.
Target group	● Name (age) *<gender randomised & age group equal to respondent>*<br>● Name (age) *<gender randomised & age group not equal to respondent (randomised)>.*<br>*Names:*<br>○ *(male first names)* Franz, Martin, Stefan, Michael, Lukas, Maximilian, Thomas, Josef, Valentin, Jakob<br>○ *(female first names)* Maria, Sabine, Daniela, Julia, Lena, Anna, Martina, Tanja, Erika, Sylvia<br>*Age:*<br>○ *(14-24 years)* 16 years, 21 years<br>○ *(25-34 years)* 28 years, 32 years<br>○ *(35-44 years)* 37 years, 43 years<br>○ *(45-54 years)* 49 years, 54 years<br>○ *(55-64 years)* 56 years, 63 years<br>○ *(65 + years)* 67 years, 72 years
Vaccination motivation (`Motiv‘)<br>● *Economic risk – sociotropic*<br>● *Economic risk - personal*<br>● *Health risk - Self-protection Recovered*<br>● *Health hazard - self-protection - severe course*<br>● *Health risk - external protection interpersonal*<br>● *Health risk - external protection collective*<br>● *Sense of community*<br>● *Self-efficacy*	● I don't want any more lockdowns! The shops and restaurants must stay open.<br>● I can't afford financially to get sick with Long Covid and not be able to work.<br>● I already had Corona and I don't want to have it again.<br>● I am afraid of a severe course of the disease. It can affect anyone.<br>● A friend of mine is a high-risk patient. I want to protect those around me.<br>● I want to protect the health system. The workload of health workers must be reduced.<br>● We should all stick together to overcome the crisis.<br>● We are not powerless in the face of the pandemic. Everyone can do something!

[1] The COVID-19 vaccine of Novavax is a protein subunit vaccine. The COVID-19 vaccine of Valneva is an inactivated whole virus vaccine. However, in public discourse, especially in Austria, both vaccines were often referred to as "inactivated COVID-19 vaccines".

Table S3.2: German version

**Attribute**	**Ausprägungen**
Virusvarianten<br>● *Decline*<br>● *No change*<br>● *Escalation*	● die weniger gefährlich sind als die derzeitige Virusvariante.<br>● die ähnlich sind wie die derzeitige Virusvariante.<br>● die gefährlicher sind als die derzeitige Virusvariante.
Art des Impfstoffs<br>● *mRNA-Impfstoffe only*<br>● *mRNA-Impfstoffe und zusätzlich Totimpfstoffe*	● mRNA-Impfstoffe: BioNtech-Pfizer, Moderna<br>● mRNA-Impfstoffe: BioNtech-Pfizer, Moderna Totimpfstoffe: Novavax[2], Valneva
Anpassung an Omikron	● verfügbar<br>● nicht verfügbar
Incentives<br>● *kostenfrei*<br>● *nicht kostenfrei*<br>● *Sachprämie*<br>● *Geldprämie*	● Die Impfung ist gratis.<br>● Die Impfung kostet 20 Euro.<br>● Für die Impfung erhalten Sie einen Einkaufsgutschein über 500 Euro als Aufwandsentschädigung.<br>● Für die Impfung erhalten Sie 500 Euro als Aufwandsentschädigung.
Zielgruppe	● Name (Alter) *<Geschlecht randomisiert & Altersgruppe gleich befragter Person>*<br>● Name (Alter) *<Geschlecht randomisiert & Altersgruppe ungleich befragte Person (randomisiert)>*<br>*Namen:*<br>○ *(männliche Vornamen)* Franz, Martin, Stefan, Michael, Lukas, Maximilian, Thomas, Josef, Valentin, Jakob<br>○ *(weibliche Vornamen)* Maria, Sabine, Daniela, Julia, Lena, Anna, Martina, Tanja, Erika, Sylvia<br>*Alter:*<br>○ *(14-24 Jahre)* 16 Jahre, 21 Jahre<br>○ *(25-34 Jahre)* 28 Jahre, 32 Jahre<br>○ *(35-44 Jahre)* 37 Jahre, 43 Jahre<br>○ *(45-54 Jahre)* 49 Jahre, 54 Jahre<br>○ *(55-64 Jahre)* 56 Jahre, 63 Jahre<br>○ *(65 + Jahre)* 67 Jahre, 72 Jahre
Impfmotivation<br>● *Wirtschaftliche Gefahr – soziotropisch*<br>● *Wirtschaftliche Gefahr – persönlich*<br>● *Gesundheitliche Gefahr - Selbstschutz Genesene*<br>● *Gesundheitliche Gefahr - Selbstschutz - schwerer Verlauf*<br>● *Gesundheitliche Gefahr - Fremdschutz interpersonal*<br>● *Gesundheitliche Gefahr - Fremdschutz kollektiv*	● Ich will keine Lockdowns mehr! Die Geschäfte und Restaurants müssen offenbleiben.<br>● Ich kann es mir finanziell nicht leisten, an Long Covid zu erkranken und nicht mehr arbeiten zu können.<br>● Ich hatte schon Corona und will es nicht schon wieder haben.<br>● Ich habe Angst vor einem schweren Krankheitsverlauf. Es kann jeden treffen.<br>● Ein Freund von mir ist Risikopatient. Ich will mein Umfeld mitschützen.<br>● Ich will das Gesundheitssystem schützen. Das Gesundheitspersonal muss entlastet werden.

[2] The COVID-19 vaccine of Novavax is a protein subunit vaccine. The COVID-19 vaccine of Valneva is an inactivated whole virus vaccine. However, in public discourse, especially in Austria, both vaccines were often referred to as "inactivated COVID-19 vaccines".

Attribute	Ausprägungen
● *Gemeinschaftsgefühl*<br><br>● *Selbstwirksamkeit*	● Wir sollten alle zusammenhalten, um die Krise gemeinsam zu meistern.<br>● Wir sind nicht machtlos gegenüber der Pandemie. Jeder kann was tun!

Table S3.3: Italian version

Attributi	Espressioni
Varianti di virus<br>● *Declino*<br>● *Nessuna variazione*<br>● *Escalation*	 ● che sono meno pericolose dell'attuale variante del virus<br>● che sono simili all'attuale variante del virus<br>● che sono più pericolose dell'attuale variante del virus
Tipo di vaccino<br>● *Solo vaccini a mRNA*<br>● *vaccini a mRNA e vaccini inattivati aggiuntivi*	 ● Vaccini a mRNA: BioNtech-Pfizer, Moderna<br>● Vaccini a mRNA: BioNtech-Pfizer, Moderna<br>Vaccini inattivati: Novavax[3], Valneva
Adattamento a Omicron	● disponibile<br>● non disponibile
Incentivi<br>● *gratuito*<br>● *non gratuito*<br>● *Premio in natura*<br>● *Premio in denaro*	 ● La vaccinazione è gratuita.<br>● La vaccinazione costa 20 euro.<br>● Per la vaccinazione riceve un buono spesa di 500 euro.<br>● Per la vaccinazione riceve 500 euro in contanti come rimborso spese.
Gruppo target	● Nome (età) *<sesso randomizzato e gruppo di età uguale a quello del rispondente>.*<br>● Nome (età) *<sesso randomizzato e gruppo di età del rispondente non uguale (randomizzato)>.*<br><br>*Nomi:*<br>○ *(nomi maschili)* Franco, Martino, Stefano, Michele, Luca, Massimiliano, Tommaso, Giuseppe, Valentino, Jacopo<br>○ *(nomi femminili)* Maria, Sabina, Daniela, Giulia, Lena, Anna, Martina, Tania, Erica, Silvia<br>*Età:*<br>○ *(14-24 anni)* 16 anni, 21 anni<br>○ *(25-34 anni)* 28 anni, 32 anni<br>○ *(35-44 anni)* 37 anni, 43 anni<br>○ *(45-54 anni)* 49 anni, 54 anni<br>○ *(55-64 anni)* 56 anni, 63 anni<br>○ *(65+ anni)* 67 anni, 72 anni
Motivazione alla vaccinazione<br>● *Rischio economico - sociotropico*	 ● Non voglio più lockdown! I negozi e i ristoranti devono rimanere aperti.

[3] The COVID-19 vaccine of Novavax is a protein subunit vaccine. The COVID-19 vaccine of Valneva is an inactivated whole virus vaccine. However, in public discourse, especially in Austria, both vaccines were often referred to as "inactivated COVID-19 vaccines".

Attributi	Espressioni
• *Rischio economico – personale*	• Non posso permettermi finanziariamente di ammalarmi di Long Covid e di non poter lavorare.
• *Pericolo per la salute - Autoprotezione convalescenti*	• Ho già avuto il Covid-19 e non voglio averlo di nuovo.
• *Pericolo per la salute - autoprotezione - decorso grave*	• Temo un decorso grave della malattia. Può colpire chiunque.
• *Pericolo per la salute - protezione degli altri*	• Un mio amico è un paziente ad alto rischio. Voglio proteggere coloro che mi circondano.
• *Pericolo per la salute - protezione per la popolazione generale*	• Voglio proteggere il sistema sanitario. Il carico di lavoro degli operatori sanitari deve essere ridotto.
• *Spirito comunitario*	• Dovremmo essere tutti uniti per superare la crisi.
• *Autoefficacia*	• Non siamo impotenti di fronte alla pandemia. Tutti possono fare qualcosa!

Supplemental File 4. Details on experiment 2 – Media communication on vaccinations

The experiment's introductory sentence reads: “In the following, we show you some made-up media reports on vaccination against COVID-19. These are fictional scenarios in each case.” Respondents were then shown two media reports at a time and were asked to answer two follow-up questions (Q3, Q4). Levels were randomly assigned with equal probabilities and repeatedly recombined, with all combinations being plausible and realistic. The procedure was repeated twice so that each respondent had to evaluate four scenarios in total.

Media Report 1	Media Report 2
TV discussion:<br>The talk show on the topic "Should we vaccinate ourselves (again)?" featured guests <Cons, 1 of 4>.	TV discussion:<br>The talk show on the topic "Should we vaccinate ourselves (again)?" featured guests <Cons, 1 of 4>.
Celebrity Newsflash:<br>< Celeb, 1 of 4>	Celebrity Newsflash:<br>< Celeb, 1 of 4>
Science:<br>A new study has investigated the incidence of Long Covid. According to the study, <LongCo, 1 in 4> suffer from Long Covid symptoms and were off work sick for more than 4 weeks. Vaccination reduces the risk of Long Covid by 50 percent.	Science:<br>A new study has investigated the incidence of Long Covid. According to the study, <LongCo, 1 in 4> suffer from Long Covid symptoms and were off work sick for more than 4 weeks. Vaccination reduces the risk of Long Covid by 50 percent.
Current Corona Rules:<br>A valid vaccination certificate will <GreenPa, 1 of 2>.	Current Corona Rules:<br>A valid vaccination certificate will <GreenPa, 1 of 2>.
Mandatory vaccination:<br><Mand, 1 of 3>	Mandatory vaccination:<br><Mand, 1 of 3>

Q3. Please read these two media reports carefully.

Based on which media report would you be more likely to trust vaccination against COVID-19? Please spontaneously choose one if neither or both media reports give an equally trustworthy impression. (Binary choice)

- Media report 1
- Media report 2

Q4. On a scale of 0 to 10, how likely is it that you would get vaccinated against COVID-19 given these media reports? (Rating scale)

- Media report 1: 0 = “would definitely not get vaccinated” to 10 “would definitely get vaccinated”
- Media report 2: 0 =” would definitely not get vaccinated” to 10 “would definitely get vaccinated”

Table S4.1: English version

Attributes	Levels
Consensus (`Cons‘)<br>• *Consensus doctors*<br><br>• *Consensus science*	• Doctors A and B., both physicians. They agreed that the vaccines are safe and effective and called on the population to vaccinate. A representative survey by the Medical Association with more than 10,000 respondents shows that 90% of doctors trust the approved vaccines.<br>• The immunologist Prof. Dr. E and the infectiologist Prof. Dr. F. Both scientists, agreed that the vaccines are safe and effective and called on the population to vaccinate. A representative survey of the professional societies for immunology and infectiology with over 1000 respondents shows that 99% of the scientists trust the approved vaccines.

**Attributes**	**Levels**
● *False Balance Doctors*<br><br>● *False Balance Scientists*	● Doctors C and D. Dr. C was of the opinion that the vaccines were safe and effective and called for vaccination. Dr. D, on the other hand, expressed doubts about the safety and effectiveness of the vaccines and did not recommend vaccination. The debate was controversial and ended without a clear outcome.<br>● The infectiologist Prof. Dr. G and the microbiologist Prof. Dr. H. Prof. Dr. G was of the opinion that the vaccines were safe and effective and called for vaccination. Prof. Dr. H, on the other hand, expressed doubts about the safety and effectiveness of the vaccines and did not recommend vaccination. The debate was controversial and ended without a clear result.
Celebrities (`Celeb‘)<br>● *Celebrity falls ill*<br>● *Celebrity refuses vaccination*<br>● *Celebrity vaccinated*<br>● *Celebrity waits for new vaccines*	● Tennis player J: "It would have been better to have had me vaccinated earlier". Due to infiltrations in the lungs after a COVID infection, J is out for the rest of the year.<br>● Football player K: "I stand by the no to vaccination". K renounces participation in the tournament because entry into the host country is currently not possible without a vaccination certificate.<br>● Singer L: "I am already vaccinated and appeal to all my fans to do the same". L is planning a big tour all over Europe in autumn.<br>● Actress M: "I'm waiting for the new vaccines". The shooting of M's new film is postponed until next year.
Long Covid (`LongCo‘)	● 1 percent of the infected<br>● 5 percent of the infected<br>● 10 per cent of the infected<br>● 20 per cent of those infected
Green passport (`GreenPa‘)	● no longer needed.<br>● needed again in many areas.
Vaccine mandate (`Mand‘)	● There is no compulsory vaccination.<br>● Compulsory vaccination applies from the age of 50 with a fine of 100 euros.<br>● Vaccination is compulsory from the age of 18 with a fine of 1500 euros.

Table S4.2: German version

Attribute	Ausprägungen
Konsens<br>● *Konsens Ärzte*<br><br>● *Konsens Wissenschaft*<br><br>● *False Balance Ärzte*	● Die Ärzte Dr. A und Dr. B. Beide Mediziner waren sich einig, dass die Impfstoffe sicher und effektiv sind, und riefen die Bevölkerung zur Impfung auf. Eine repräsentative Umfrage der Ärztekammer mit über 10.000 Befragten zeigt, dass 90% der Ärztinnen und Ärzte den zugelassenen Impfstoffen vertrauen.<br>● Der Immunologe Prof. Dr. E und der Infektiologe Prof. Dr. F. Beide Wissenschaftler waren sich einig, dass die Impfstoffe sicher und effektiv sind, und riefen die Bevölkerung zur Impfung auf. Eine repräsentative Befragung der Fachgesellschaften für Immunologie und Infektiologie mit über 1000 Befragten zeigt, dass 99% der Wissenschaftlerinnen und Wissenschaftlern den zugelassenen Impfstoffen vertrauen.

Attribute	Ausprägungen
● *False Balance Wissenschaftler*	● Die Ärzte Dr. C und Dr. D. Dr. C vertrat die Meinung, dass die Impfstoffe sicher und effektiv seien und rief zur Impfung auf. Dr. D hingegen äußerte Zweifel in Bezug auf die Sicherheit und Wirksamkeit der Impfstoffe und sprach keine Impfempfehlung aus. Die Debatte war kontrovers und endete ohne ein klares Ergebnis.<br>● Der Infektiologe Prof. Dr. G und der Mikrobiologe Prof. Dr. H. Prof Dr. G vertrat die Meinung, dass die Impfstoffe sicher und effektiv seien und rief zur Impfung auf. Prof. Dr. H hingegen äußerte Zweifel in Bezug auf die Sicherheit und Wirksamkeit der Impfstoffe und sprach keine Impfempfehlung aus. Die Debatte war kontrovers und endete ohne ein klares Ergebnis.
Promis<br>● *Promi erkrankt*<br>● *Promi verweigert Impfung*<br>● *Promi geimpft*<br>● *Promi wartet auf neue Impfstoffe*	● Tennisspielerin J: “Es wäre besser gewesen, mich früher impfen zu lassen”. Aufgrund von Infiltrationen in der Lunge nach einer COVID-Infektion fällt J für den Rest des Jahres aus.<br>● Fußballspieler K: “Ich bleibe beim Nein zur Impfung”. K verzichtet auf die Teilnahme am Turnier, da die Einreise ins Austragungsland ohne Impfzertifikat derzeit nicht möglich ist.<br>● Sänger L: “Ich bin schon geimpft und appelliere an all meine Fans, das auch zu tun”. L plant eine große Tournee im Herbst durch ganz Europa.<br>● Schauspielerin M: “Ich warte auf die neuen Impfstoffe”. Die Dreharbeiten für M’s neuen Film verschieben sich auf das nächste Jahr.
Long Covid	● 1 Prozent der Infizierten<br>● 5 Prozent der Infizierten<br>● 10 Prozent der Infizierten<br>● 20 Prozent der Infizierten
Grüner Pass	● nicht mehr benötigt.<br>● in vielen Bereichen wieder benötigt.
Impfpflicht	● Es gilt keine Impfpflicht.<br>● Es gilt eine Impfpflicht ab 50 Jahre mit einer Geldstrafe von 100 Euro.<br>● Es gilt eine Impfpflicht ab 18 Jahre mit einer Geldstrafe von 1500 Euro.

Table S4.3: Italian version

Attributi	Espressioni
Consenso<br>● *Medici di consenso*<br>● *Scienza del consenso*<br>● *Falso Equilibrio Medici*	● i medici Dr. A e Dr. B. Entrambi i medici concordano sul fatto che i vaccini sono sicuri ed efficaci e invitano la popolazione a vaccinarsi. Un sondaggio rappresentativo dell'Associazione Medica con più di 10.000 intervistati mostra che il 90% dei medici si fida dei vaccini approvati.<br>● l'immunologo Prof. Dr. E e l'infettivologo Prof. Dr. F. Entrambi gli scienziati sono d’accordo che i vaccini sono sicuri ed efficaci e hanno invitato la popolazione a vaccinarsi. Un sondaggio rappresentativo delle società professionali di immunologia e infettivologia con oltre 1.000 intervistati mostra che il 99% degli scienziati si fida dei vaccini approvati.<br>● i medici Dr. I e Dr. J. Il dottor I era del parere che i vaccini fossero sicuri ed efficaci e ha invitato a vaccinarsi. Il dottor J, invece, ha espresso dubbi sulla sicurezza e sull'efficacia dei vaccini e non ha consigliato la vaccinazione. Il dibattito era controverso e si è concluso senza un esito chiaro.<br>● l'infettivologo Prof. Dr. G e il microbiologo Prof. Dr. H. Il Professor G era del parere che i vaccini fossero sicuri ed efficaci e ha invitato alla vaccinazione. Il

Attributi	Espressioni
• *Scienziati in falso equilibrio*	Professor H, invece, ha espresso dubbi sulla sicurezza e sull'efficacia dei vaccini e non ha consigliato la vaccinazione. Il dibattito era controverso e si è concluso senza un risultato chiaro.
Celebrità<br>• *Una celebrità si ammala*<br>• *Una celebrità rifiuta la vaccinazione*<br>• *Celebrità vaccinate*<br>• *Celebrità in attesa di nuovi vaccini*	• Il tennista J: "Sarebbe stato meglio farmi vaccinare prima". A causa di danni ai polmoni dopo un'infezione da Covid-19, J é fuori per il resto dell'anno.<br>• Il giocatore di calcio K: "Sostengo il no alla vaccinazione". K rinuncia a partecipare al torneo perché l'ingresso nel Paese ospitante non é attualmente possibile senza un certificato di vaccinazione.<br>• La cantante L: "Sono già vaccinata e chiedo a tutti i miei fan di fare lo stesso". L ha in programma un grande tour in tutta Europa in autunno.<br>• Attrice M: "Aspetto i nuovi vaccini". Le riprese del nuovo film di M sono rimandate al prossimo anno.
Long Covid	• 5 percento degli infetti<br>• 10 percento degli infetti<br>• 20 percento degli infetti<br>• L'1 percento degli infetti
Passaporto verde	• non è più necessario.<br>• necessario in molti settori.
Vaccinazione obbligatoria	• Non esiste una vaccinazione obbligatoria.<br>• La vaccinazione obbligatoria si applica a partire dai 50 anni di età con una multa di 100 euro.<br>• La vaccinazione è obbligatoria a partire dai 18 anni con una multa di 1.500 euro.

Supplemental File 5. Effects on the evaluation of the campaign by country (experiment 1)

We calculated Average Marginal Component Effects (AMCEs). Data are presented as AMCE estimates +/- 95% confidence intervals. Exact p-values are shown in column 7. The Bonferroni corrected significance level is 0.0031. Significant p-values are marked with (in bold and with a *) and without Bonferroni correction (in bold).

Sub-group	Attribute	Levels	Est.	SE	z	p	95% Confidence interval	
							lower	upper
AT	Virus_variant	No_change	0					
		Escalation	0.02148	0.01078	1.99256	**0.04631**	0.00035	0.04261
		Decline	-0.04928	0.01063	-4.63419	**<0.00001***	-0.07012	-0.02844
	Vaccines	mRNA_only	0					
		mRNA+Inactive	0.02754	0.00869	3.17065	**0.00152***	0.01052	0.04457
	Omicron_adapted	Not_adapted	0					
		Adapted	0.08013	0.00877	9.13218	**<0.00001***	0.06293	0.09732
	Incentives	Free	0					
		20_Euro_fee	-0.17618	0.01245	-14.15312	**<0.00001***	-0.20058	-0.15178
		500_Euro_cash	0.04008	0.01268	3.16179	**0.00157***	0.01523	0.06492
		500_Euro_voucher	0.01121	0.01268	0.88416	0.37661	-0.01364	0.03606
	Motivation	Risk_re-infection	0					
		Risk_unable_to_work	0.00839	0.01736	0.48325	0.62892	-0.02564	0.04242
		Risk_severe_disease	0.05191	0.01731	2.99940	**0.00271***	0.01799	0.08584
		Community_spirit	0.04178	0.01760	2.37430	**0.01758**	0.00729	0.07626
		Protect_friends	0.03618	0.01739	2.08088	**0.03744**	0.00210	0.07026
		Self-efficacy	0.03827	0.01730	2.21221	**0.02695**	0.00436	0.07218
		Risk_lockdown	0.03097	0.01754	1.76515	0.07754	-0.00342	0.06535
		Protect_health_system	0.04387	0.01746	2.51257	**0.01199**	0.00965	0.07808
IT	Virus_variant	No_change	0					
		Escalation	0.00561	0.01092	0.51396	0.60728	-0.01579	0.02701
		Decline	-0.00796	0.01076	-0.73971	0.45948	-0.02904	0.01313
	Vaccines	mRNA_only	0					
		mRNA+Inactive	0.00588	0.00898	0.65469	0.51266	-0.01172	0.02347
	Omicron_adapted	Not_adapted	0					
		Adapted	0.07371	0.00890	8.28187	**<0.00001***	0.05626	0.09115
	Incentives	Free	0					
		20_Euro_fee	-0.14653	0.01232	-11.89637	**<0.00001***	-0.17067	-0.12239
		500_Euro_cash	-0.01637	0.01280	-1.27911	0.20086	-0.04145	0.00871
		500_Euro_voucher	-0.01567	0.01268	-1.23578	0.21654	-0.04051	0.00918
	Motivation	Risk_re-infection	0					
		Risk_unable_to_work	0.00258	0.01769	0.14565	0.88420	-0.03210	0.03725
		Risk_severe_disease	0.01439	0.01781	0.80789	0.41916	-0.02052	0.04931
		Community_spirit	0.03924	0.01754	2.23798	**0.02522**	0.00488	0.07361
		Protect_friends	0.04365	0.01776	2.45810	**0.01397**	0.00884	0.07845
		Self-efficacy	0.03644	0.01777	2.05106	**0.04026**	0.00162	0.07126
		Risk_lockdown	0.03642	0.01755	2.07557	**0.03793**	0.00203	0.07082
		Protect_health_system	0.05150	0.01770	2.91049	**0.00361**	0.01682	0.08619

Notes: Estimates are Average Marginal Component Effects (AMCEs).

Supplemental File 6. Effects on the likelihood to get vaccinated by country (experiment 1)

We calculated Average Marginal Component Effects (AMCEs). Data are presented as AMCE estimates +/- 95% confidence intervals. Exact p-values are shown in column 7. The Bonferroni corrected significance level is 0.0031. Significant p-values are marked with (in bold and with a *) and without Bonferroni correction (in bold).

Sub-group	Attribute	Levels	Est.	SE	z	p	95% Confidence interval	
							lower	upper
AT	Virus_variant	No_change	0					
		Escalation	0.12837	0.07850	1.63531	0.10198	-0.02549	0.28223
		Decline	-0.11292	0.07621	-1.48181	0.13839	-0.26228	0.03644
	Vaccines	mRNA_only	0					
		mRNA+Inactive	-0.04691	0.06549	-0.71630	0.47381	-0.17527	0.08145
	Omicron_adapted	Not_adapted	0					
		Adapted	0.25619	0.06691	3.82883	**0.00013***	0.12505	0.38733
	Incentives	Free	0					
		20_Euro_fee	-0.50484	0.09353	-5.39740	**<0.00001***	-0.68817	-0.32152
		500_Euro_cash	0.30662	0.09180	3.34003	**0.00084***	0.12669	0.48654
		500_Euro_voucher	0.38445	0.09484	4.05380	**0.00005***	0.19857	0.57033
	Motivation	Risk_re-infection	0					
		Risk_unable_to_work	0.01050	0.12866	0.08158	0.93498	-0.24167	0.26266
		Risk_severe_disease	0.19628	0.13055	1.50346	0.13272	-0.05960	0.45216
		Community_spirit	0.09315	0.12816	0.72678	0.46736	-0.15805	0.34434
		Protect_friends	-0.10693	0.13147	-0.81330	0.41604	-0.36462	0.15076
		Self-efficacy	0.05195	0.13122	0.39590	0.69218	-0.20524	0.30915
		Risk_lockdown	0.12793	0.12765	1.00218	0.31626	-0.12226	0.37811
		Protect_health_system	0.03043	0.13096	0.23237	0.81625	-0.22624	0.28710
IT	Virus_variant	No_change	0					
		Escalation	-0.04795	0.06546	-0.73260	0.46380	-0.17625	0.08034
		Decline	-0.09072	0.06660	-1.36228	0.17311	-0.22125	0.03980
	Vaccines	mRNA_only	0					
		mRNA+Inactive	0.00983	0.05368	0.18312	0.85470	-0.09538	0.11504
	Omicron_adapted	Not_adapted	0					
		Adapted	0.11514	0.05525	2.08415	**0.03715**	0.00686	0.22343
	Incentives	Free	0					
		20_Euro_fee	-0.64739	0.07823	-8.27507	**<0.00001***	-0.80073	-0.49406
		500_Euro_cash	0.06529	0.07927	0.82368	0.41012	-0.09008	0.22067
		500_Euro_voucher	0.12289	0.07651	1.60633	0.10820	-0.02706	0.27284
	Motivation	Risk_re-infection	0					
		Risk_unable_to_work	-0.10267	0.10754	-0.95472	0.33972	-0.31345	0.10811
		Risk_severe_disease	0.01631	0.10863	0.15012	0.88067	-0.19660	0.22921
		Community_spirit	0.09134	0.10987	0.83130	0.40580	-0.12401	0.30669
		Protect_friends	-0.06974	0.10799	-0.64580	0.51841	-0.28140	0.14192
		Self-efficacy	-0.04882	0.10640	-0.45880	0.64638	-0.25736	0.15973
		Risk_lockdown	-0.00264	0.10679	-0.02472	0.98028	-0.21194	0.20666
		Protect_health_system	0.09911	0.10877	0.91118	0.36220	-0.11408	0.31230

Notes: Estimates are Average Marginal Component Effects (AMCEs).

Supplemental File 7. Effects on the evaluation of the campaign by vaccination status (experiment 1)

We calculated Average Marginal Component Effects (AMCEs). Data are presented as AMCE estimates +/- 95% confidence intervals. Exact p-values are shown in column 7. The Bonferroni corrected significance level is 0.0031. Significant p-values are marked with (in bold and with a *) and without Bonferroni correction (in bold).

Sub-group	Attribute	Levels	Est.	SE	z	p	95% Confidence interval	
							lower	upper
Not	Virus_variant	No_change	0					
		Escalation	-0.01091	0.01548	-0.70450	0.48112	-0.04126	0.01944
		Decline	0.00129	0.01539	0.08357	0.93340	-0.02889	0.03146
	Vaccines	mRNA_only	0					
		mRNA+Inactive	0.04483	0.01289	3.47692	**0.00051***	0.01956	0.07011
	Omicron_adapted	Not_adapted	0					
		Adapted	0.03528	0.01280	2.75591	**0.00585**	0.01019	0.06036
	Incentives	Free	0					
		20_Euro_fee	-0.09397	0.01798	-5.22532	**<0.00001***	-0.12921	-0.05872
		500_Euro_cash	-0.03598	0.01789	-2.01166	**0.04426**	-0.07104	-0.00092
		500_Euro_voucher	-0.03283	0.01783	-1.84122	0.06559	-0.06778	0.00212
	Motivation	Risk_re-infection	0					
		Risk_unable_to_work	0.02856	0.02461	1.16014	0.24599	-0.01969	0.07680
		Risk_severe_disease	0.02069	0.02451	0.84446	0.39841	-0.02734	0.06873
		Community_spirit	0.06948	0.02452	2.83415	**0.00459***	0.02143	0.11753
		Protect_friends	0.05587	0.02454	2.27655	**0.02281**	0.00777	0.10397
		Self-efficacy	0.04753	0.02452	1.93867	0.05254	-0.00052	0.09558
		Risk_lockdown	0.05230	0.02511	2.08281	**0.03727**	0.00308	0.10151
		Protect_health_system	0.05215	0.02459	2.12021	**0.03399**	0.00394	0.10035
1 or 2	Virus_variant	No_change	0					
		Escalation	0.05157	0.02027	2.54379	**0.01097**	0.01184	0.09130
		Decline	-0.01863	0.01983	-0.93962	0.34741	-0.05749	0.02023
	Vaccines	mRNA_only	0					
		mRNA+Inactive	0.03714	0.01633	2.27399	**0.02297**	0.00513	0.06915
	Omicron_adapted	Not_adapted	0					
		Adapted	0.05556	0.01612	3.44766	**0.00057***	0.02397	0.08714
	Incentives	Free	0					
		20_Euro_fee	-0.15572	0.02312	-6.73551	**<0.00001***	-0.20104	-0.11041
		500_Euro_cash	0.10531	0.02405	4.37903	**0.00001***	0.05817	0.15244
		500_Euro_voucher	0.04374	0.02406	1.81806	0.06906	-0.00341	0.09090
	Motivation	Risk_re-infection	0					
		Risk_unable_to_work	0.00299	0.03459	0.08638	0.93116	-0.06481	0.07079
		Risk_severe_disease	-0.01688	0.03419	-0.49379	0.62145	-0.08390	0.05013
		Community_spirit	-0.01836	0.03442	-0.53324	0.59386	-0.08583	0.04911
		Protect_friends	0.01445	0.03347	0.43175	0.66593	-0.05115	0.08005
		Self-efficacy	0.02422	0.03497	0.69262	0.48855	-0.04432	0.09277
		Risk_lockdown	0.00870	0.03413	0.25505	0.79868	-0.05819	0.07560
		Protect_health_system	0.01922	0.03420	0.56192	0.57417	-0.04781	0.08625
≥3	Virus_variant	No_change	0					

Sub-group	Attribute	Levels	Est.	SE	z	p	95% Confidence interval	
							lower	upper
		Escalation	0.01540	0.00979	1.57349	0.11560	-0.00378	0.03458
		Decline	-0.04293	0.00964	-4.45250	**0.00001***	-0.06183	-0.02403
	Vaccines	mRNA_only	0					
		mRNA+Inactive	0.00105	0.00789	0.13335	0.89391	-0.01440	0.01651
	Omicron_adapted	Not_adapted	0.00000					
		Adapted	0.10067	0.00795	12.66216	**<0.00001***	0.08509	0.11625
	Incentives	Free	0.00000					
		20_Euro_fee	-0.19224	0.01104	-17.41449	**<0.00001***	-0.21388	-0.17061
		500_Euro_cash	0.01169	0.01152	1.01486	0.31017	-0.01089	0.03427
		500_Euro_voucher	-0.00062	0.01147	-0.05365	0.95722	-0.02310	0.02187
	Motivation	Risk_re-infection	0.00000					
		Risk_unable_to_work	-0.00395	0.01576	-0.25036	0.80231	-0.03484	0.02695
		Risk_severe_disease	0.05132	0.01583	3.24147	**0.00119***	0.02029	0.08234
		Community_spirit	0.04491	0.01584	2.83607	**0.00457**	0.01387	0.07595
		Protect_friends	0.04201	0.01598	2.62921	**0.00856**	0.01069	0.07333
		Self-efficacy	0.03812	0.01574	2.42233	**0.01542**	0.00728	0.06896
		Risk_lockdown	0.03322	0.01570	2.11596	**0.03435**	0.00245	0.06399
		Protect_health_system	0.05355	0.01584	3.38183	**0.00072***	0.02252	0.08459

Notes: Estimates are Average Marginal Component Effects (AMCEs).

Supplemental File 8. Effects on the likelihood to get vaccinated by vaccination status (experiment 1)

We calculated Average Marginal Component Effects (AMCEs). Data are presented as AMCE estimates +/- 95% confidence intervals. Exact p-values are shown in column 7. The Bonferroni corrected significance level is 0.0031. Significant p-values are marked with (in bold and with a *) and without Bonferroni correction (in bold).

Sub-group	Attribute	Levels	Est.	SE	z	p	95% Confidence interval	
							lower	upper
Not	Virus_variant	No_change	0					
		Escalation	-0.07368	0.09792	-0.75247	0.45177	-0.26561	0.11824
		Decline	-0.09389	0.10076	-0.93178	0.35145	-0.29138	0.10360
	Vaccines	mRNA_only	0					
		mRNA+Inactive	-0.07497	0.08201	-0.91414	0.36064	-0.23570	0.08577
	Omicron_adapted	Not_adapted	0					
		Adapted	-0.00181	0.08171	-0.02213	0.98235	-0.16196	0.15834
	Incentives	Free	0					
		20_Euro_fee	-0.02668	0.11388	-0.23424	0.81480	-0.24988	0.19653
		500_Euro_cash	0.15437	0.11490	1.34351	0.17911	-0.07083	0.37958
		500_Euro_voucher	0.08945	0.12028	0.74368	0.45707	-0.14629	0.32518
	Motivation	Risk_re-infection	0					
		Risk_unable_to_work	-0.11694	0.15892	-0.73581	0.46185	-0.42843	0.19455
		Risk_severe_disease	-0.02317	0.16328	-0.14192	0.88714	-0.34319	0.29685
		Community_spirit	0.34251	0.16506	2.07513	**0.03797**	0.01901	0.66601
		Protect_friends	0.01827	0.16462	0.11098	0.91163	-0.30439	0.34093
		Self-efficacy	-0.07525	0.16166	-0.46550	0.64157	-0.39210	0.24159
		Risk_lockdown	0.00577	0.16198	0.03559	0.97161	-0.31170	0.32323
		Protect_health_system	0.01671	0.15842	0.10548	0.91599	-0.29379	0.32722
1 or 2	Virus_variant	No_change	0					
		Escalation	0.10051	0.12267	0.81934	0.41259	-0.13992	0.34094
		Decline	-0.07083	0.12253	-0.57803	0.56324	-0.31099	0.16933
	Vaccines	mRNA_only	0					
		mRNA+Inactive	0.20805	0.10610	1.96093	**0.04989**	0.00010	0.41600
	Omicron_adapted	Not_adapted	0					
		Adapted	0.22218	0.10721	2.07238	**0.03823**	0.01205	0.43231
	Incentives	Free	0					
		20_Euro_fee	-0.43569	0.14864	-2.93113	**0.00338**	-0.72703	-0.14436
		500_Euro_cash	0.72170	0.14923	4.83619	**<0.00001***	0.42922	1.01419
		500_Euro_voucher	0.67009	0.15138	4.42652	**0.00001***	0.37339	0.96679
	Motivation	Risk_re-infection	0					
		Risk_unable_to_work	-0.15762	0.21244	-0.74196	0.45811	-0.57399	0.25875
		Risk_severe_disease	0.02693	0.21613	0.12459	0.90085	-0.39669	0.45054
		Community_spirit	-0.06403	0.21579	-0.29671	0.76669	-0.48697	0.35892
		Protect_friends	-0.23059	0.20286	-1.13670	0.25567	-0.62818	0.16700
		Self-efficacy	-0.14123	0.21358	-0.66126	0.50844	-0.55985	0.27738
		Risk_lockdown	0.12467	0.21650	0.57587	0.56470	-0.29965	0.54900
		Protect_health_system	0.24894	0.22490	1.10689	0.26834	-0.19186	0.68975
≥3	Virus_variant	No_change	0					

Sub-group	Attribute	Levels	Est.	SE	z	p	95% Confidence interval	
							lower	upper
		Escalation	0.12168	0.05854	2.07857	**0.03766**	0.00694	0.23641
		Decline	-0.09803	0.05631	-1.74092	0.08170	-0.20840	0.01233
	Vaccines	mRNA_only	0					
		mRNA+Inactive	-0.02184	0.04743	-0.46045	0.64520	-0.11481	0.07113
	Omicron_adapted	Not_adapted	0					
		Adapted	0.27937	0.04978	5.61235	**<0.00001***	0.18181	0.37693
	Incentives	Free	0					
		20_Euro_fee	-0.79465	0.07162	-11.09570	**<0.00001***	-0.93502	-0.65428
		500_Euro_cash	0.10122	0.06949	1.45655	0.14524	-0.03498	0.23742
		500_Euro_voucher	0.19444	0.06789	2.86395	**0.00418**	0.06137	0.32750
	Motivation	Risk_re-infection	0					
		Risk_unable_to_work	-0.04325	0.09454	-0.45741	0.64738	-0.22855	0.14206
		Risk_severe_disease	0.10482	0.09553	1.09723	0.27254	-0.08242	0.29206
		Community_spirit	0.05354	0.09602	0.55752	0.57717	-0.13467	0.24174
		Protect_friends	-0.06231	0.09946	-0.62646	0.53101	-0.25725	0.13263
		Self-efficacy	0.06805	0.09559	0.71192	0.47651	-0.11930	0.25540
		Risk_lockdown	0.04964	0.09523	0.52125	0.60219	-0.13701	0.23629
		Protect_health_system	0.06947	0.09715	0.71510	0.47455	-0.12093	0.25987

Notes: Estimates are Average Marginal Component Effects (AMCEs).

Supplemental File 9. Effects on trust in the vaccine by country (experiment 2)

We calculated Average Marginal Component Effects (AMCEs). Data are presented as AMCE estimates +/- 95% confidence intervals. Exact p-values are shown in column 7. The Bonferroni corrected significance level is 0.0041. Significant p-values are marked with (in bold and with a *) and without Bonferroni correction (in bold).

Sub-group	Attribute	Levels	Est.	SE	z	p	95% Confidence interval	
							lower	upper
AT	Consensus	Consensus_scientists	0					
		Consensus_physicians	-0.02507	0.01233	-2.03265	**0.04209**	-0.04924	-0.00090
		Dissensus_scientists	-0.07042	0.01238	-5.68660	**<0.00001***	-0.09469	-0.04615
		Dissensus_physicians	-0.07996	0.01271	-6.28968	**<0.00001***	-0.10487	-0.05504
	Celebrity	Refuses_vaccine	0					
		Endorses_vaccine	0.05681	0.01270	4.47460	**0.00001***	0.03193	0.08170
		Waits_for_new_vaccine	0.01409	0.01226	1.14975	0.25025	-0.00993	0.03811
		Infected_regrets_hesitancy	0.05858	0.01221	4.79975	**<0.00001***	0.03466	0.08251
	Long_COVID	1_percent	0					
		5_percent	0.02169	0.01228	1.76637	0.07733	-0.00238	0.04576
		10_percent	0.02267	0.01228	1.84683	0.06477	-0.00139	0.04674
		20_percent	0.03266	0.01243	2.62615	**0.00864**	0.00828	0.05703
	Green_pass	Not_needed	0					
		Needed	0.02365	0.00900	2.62733	**0.00861**	0.00601	0.04130
	Vaccine_mandate	No	0					
		Age_50+_fine_100€	-0.13674	0.01101	-12.41654	**<0.00001***	-0.15832	-0.11515
		Age_18+_fine_1500€	-0.18895	0.01141	-16.55585	**<0.00001***	-0.21131	-0.16658
IT	Consensus	Consensus_scientists	0					
		Consensus_physicians	-0.01947	0.01288	-1.51157	0.13064	-0.04472	0.00578
		Dissensus_scientists	-0.05497	0.01264	-4.34930	**0.00001***	-0.07974	-0.03020
		Dissensus_physicians	-0.05804	0.01276	-4.54954	**0.00001***	-0.08304	-0.03304
	Celebrity	Refuses_vaccine	0					
		Endorses_vaccine	0.05156	0.01268	4.06782	**0.00005***	0.02672	0.07640
		Waits_for_new_vaccine	0.03305	0.01248	2.64774	**0.00810**	0.00859	0.05752
		Infected_regrets_hesitancy	0.05350	0.01266	4.22502	**0.00002***	0.02868	0.07832
	Long_COVID	1_percent	0					
		5_percent	0.01804	0.01255	1.43764	0.15054	-0.00656	0.04264
		10_percent	-0.00962	0.01233	-0.77984	0.43549	-0.03378	0.01455
		20_percent	0.01861	0.01249	1.49010	0.13620	-0.00587	0.04308
	Green_pass	Not_needed	0					
		Needed	-0.01002	0.00903	-1.11024	0.26690	-0.02772	0.00767
	Vaccine_mandate	No	0					
		Age_50+_fine_100€	-0.01775	0.01106	-1.60488	0.10852	-0.03943	0.00393
		Age_18+_fine_1500€	-0.06450	0.01130	-5.70825	**<0.00001***	-0.08665	-0.04235

Notes: Estimates are Average Marginal Component Effects (AMCEs).

Supplemental File 10. Effects on the likelihood to get vaccinated by country (experiment 2)

We calculated Average Marginal Component Effects (AMCEs). Data are presented as AMCE estimates +/- 95% confidence intervals. Exact p-values are shown in column 7. The Bonferroni corrected significance level is 0.0041. Significant p-values are marked with (in bold and with a *) and without Bonferroni correction (in bold).

Sub-group	Attribute	Levels	Est.	SE	z	p	95% Confidence interval	
							lower	upper
AT	Consensus	Consensus_scientists	0					
		Consensus_physicians	0.21534	0.09099	2.36670	**0.01795**	0.03701	0.39368
		Dissensus_scientists	0.08434	0.09252	0.91165	0.36195	-0.09699	0.26567
		Dissensus_physicians	0.08176	0.09302	0.87894	0.37943	-0.10056	0.26408
	Celebrity	Refuses_vaccine	0					
		Endorses_vaccine	0.11123	0.09097	1.22268	0.22145	-0.06707	0.28952
		Waits_for_new_vaccine	0.03908	0.08939	0.43723	0.66194	-0.13612	0.21429
		Infected_regrets_hesitancy	0.13731	0.09111	1.50716	0.13177	-0.04125	0.31588
	Long_COVID	1_percent	0					
		5_percent	0.13952	0.09251	1.50812	0.13152	-0.04180	0.32084
		10_percent	0.02867	0.09061	0.31644	0.75167	-0.14891	0.20626
		20_percent	0.21252	0.09299	2.28552	**0.02228**	0.03027	0.39477
	Green_pass	Not_needed	0					
		Needed	0.10520	0.06427	1.63683	0.10167	-0.02077	0.23116
	Vaccine_mandate	No	0					
		Age_50+_fine_100€	-0.02404	0.07911	-0.30388	0.76122	-0.17908	0.13101
		Age_18+_fine_1500€	-0.00225	0.08007	-0.02810	0.97758	-0.15919	0.15469
IT	Consensus	Consensus_scientists	0					
		Consensus_physicians	0.02092	0.07422	0.28191	0.77802	-0.12455	0.16639
		Dissensus_scientists	-0.10468	0.07478	-1.39992	0.16154	-0.25124	0.04188
		Dissensus_physicians	-0.17550	0.07458	-2.35317	**0.01861**	-0.32167	-0.02932
	Celebrity	Refuses_vaccine	0					
		Endorses_vaccine	0.13838	0.07520	1.84000	0.06577	-0.00902	0.28577
		Waits_for_new_vaccine	0.03869	0.07608	0.50855	0.61107	-0.11042	0.18780
		Infected_regrets_hesitancy	0.14957	0.07423	2.01499	0.04391	0.00408	0.29506
	Long_COVID	1_percent	0					
		5_percent	-0.05572	0.07403	-0.75263	0.45167	-0.20082	0.08938
		10_percent	0.00061	0.07543	0.00807	0.99356	-0.14722	0.14844
		20_percent	-0.00616	0.07286	-0.08449	0.93267	-0.14895	0.13664
	Green_pass	Not_needed	0					
		Needed	-0.02977	0.05325	-0.55910	0.57610	-0.13415	0.07460
	Vaccine_mandate	No	0					
		Age_50+_fine_100€	-0.01946	0.06480	-0.30037	0.76390	-0.14647	0.10754
		Age_18+_fine_1500€	0.02937	0.06656	0.44118	0.65908	-0.10109	0.15982

Notes: Estimates are Average Marginal Component Effects (AMCEs).

Supplemental File 11. Effects on trust in the vaccine by vaccination status (experiment 2)

We calculated Average Marginal Component Effects (AMCEs). Data are presented as AMCE estimates +/- 95% confidence intervals. Exact p-values are shown in column 7. The Bonferroni corrected significance level is 0.0041. Significant p-values are marked with (in bold and with a *) and without Bonferroni correction (in bold).

Sub-group	Attribute	Levels	Est.	SE	z	p	95% Confidence interval	
							lower	upper
Not	Consensus	Consensus_scientists	0					
		Consensus_physicians	0.00414	0.01777	0.23290	0.81584	-0.03069	0.03896
		Dissensus_scientists	0.01052	0.01720	0.61181	0.54066	-0.02319	0.04423
		Dissensus_physicians	0.01980	0.01739	1.13852	0.25490	-0.01429	0.05389
	Celebrity	Refuses_vaccine	0					
		Endorses_vaccine	-0.03725	0.01767	-2.10824	**0.03501**	-0.07188	-0.00262
		Waits_for_new_vaccine	-0.00832	0.01735	-0.47969	0.63145	-0.04232	0.02568
		Infected_regrets_hesitancy	-0.02117	0.01721	-1.23048	0.21852	-0.05490	0.01255
	Long_COVID	1_percent	0					
		5_percent	0.01820	0.01739	1.04655	0.29531	-0.01588	0.05228
		10_percent	-0.01515	0.01694	-0.89401	0.37132	-0.04836	0.01806
		20_percent	0.01035	0.01762	0.58743	0.55691	-0.02419	0.04489
	Green_pass	Not_needed	0					
		Needed	-0.05724	0.01258	-4.54910	**0.00001***	-0.08191	-0.03258
	Vaccine_mandate	No	0					
		Age_50+_fine_100€	-0.09150	0.01522	-6.01030	**<0.00001***	-0.12134	-0.06166
		Age_18+_fine_1500€	-0.19141	0.01539	-12.44067	**<0.00001***	-0.22156	-0.16125
1 or 2	Consensus	Consensus_scientists	0					
		Consensus_physicians	-0.01146	0.02412	-0.47502	0.63477	-0.05874	0.03582
		Dissensus_scientists	-0.05056	0.02398	-2.10889	**0.03495**	-0.09755	-0.00357
		Dissensus_physicians	-0.03160	0.02477	-1.27578	0.20203	-0.08015	0.01695
	Celebrity	Refuses_vaccine	0					
		Endorses_vaccine	0.02194	0.02297	0.95540	0.33938	-0.02307	0.06695
		Waits_for_new_vaccine	0.00150	0.02307	0.06487	0.94828	-0.04372	0.04671
		Infected_regrets_hesitancy	0.01858	0.02282	0.81448	0.41537	-0.02614	0.06330
	Long_COVID	1_percent	0					
		5_percent	0.03087	0.02440	1.26525	0.20578	-0.01695	0.07868
		10_percent	-0.01203	0.02347	-0.51248	0.60831	-0.05802	0.03397
		20_percent	0.00808	0.02402	0.33659	0.73642	-0.03899	0.05515
	Green_pass	Not_needed	0					
		Needed	-0.01378	0.01724	-0.79941	0.42405	-0.04757	0.02001
	Vaccine_mandate	No	0					
		Age_50+_fine_100€	-0.11380	0.02118	-5.37254	**<0.00001***	-0.15532	-0.07229
		Age_18+_fine_1500€	-0.16697	0.02207	-7.56559	**<0.00001***	-0.21023	-0.12372
≥3	Consensus	Consensus_scientists	0					
		Consensus_physicians	-0.03542	0.01140	-3.10627	**0.00189***	-0.05777	-0.01307
		Dissensus_scientists	-0.09580	0.01141	-8.39915	**<0.00001***	-0.11816	-0.07345
		Dissensus_physicians	-0.11558	0.01151	-10.04156	**<0.00001***	-0.13814	-0.09302
	Celebrity	Refuses_vaccine	0					

Sub-group	Attribute	Levels	Est.	SE	z	p	95% Confidence interval	
							lower	upper
		Endorses_vaccine	0.10207	0.01160	8.80144	**<0.00001***	0.07934	0.12480
		Waits_for_new_vaccine	0.04370	0.01125	3.88490	**0.00010***	0.02165	0.06575
		Infected_regrets_hesitancy	0.09873	0.01134	8.70474	**>0.00001***	0.07650	0.12096
	Long_COVID	1_percent	0					
		5_percent	0.01777	0.01113	1.59552	0.11060	-0.00406	0.03959
		10_percent	0.02081	0.01119	1.85948	0.06296	-0.00112	0.04275
		20_percent	0.03559	0.01122	3.17037	**0.00152***	0.01359	0.05759
	Green_pass	Not_needed	0					
		Needed	0.03957	0.00814	4.85906	**<0.00001***	0.02361	0.05553
	Vaccine_mandate	No	0					
		Age_50+_fine_100€	-0.06248	0.01013	-6.16761	**<0.00001***	-0.08233	-0.04262
		Age_18+_fine_1500€	-0.08951	0.01042	-8.59330	**<0.00001***	-0.10992	-0.06909

Notes: Estimates are Average Marginal Component Effects (AMCEs).

Supplemental File 12. Effects on the likelihood to get vaccinated by vaccination status (experiment 2)

We calculated Average Marginal Component Effects (AMCEs). Data are presented as AMCE estimates +/- 95% confidence intervals. Exact p-values are shown in column 7. The Bonferroni corrected significance level is 0.0041. Significant p-values are marked with (in bold and with a *) and without Bonferroni correction (in bold).

Sub-group	Attribute	Levels	Est.	SE	z	p	95% Confidence interval	
							lower	upper
Not	Consensus	Consensus_scientists	0					
		Consensus_physicians	0.15903	0.11419	1.39274	0.16370	-0.06477	0.38284
		Dissensus_scientists	0.14788	0.11831	1.24992	0.21133	-0.08401	0.37977
		Dissensus_physicians	-0.01977	0.11644	-0.16982	0.86515	-0.24799	0.20844
	Celebrity	Refuses_vaccine	0					
		Endorses_vaccine	-0.07428	0.11671	-0.63647	0.52447	-0.30302	0.15446
		Waits_for_new_vaccine	0.09549	0.11341	0.84196	0.39981	-0.12679	0.31776
		Infected_regrets_hesitancy	0.08271	0.11362	0.72795	0.46664	-0.13998	0.30540
	Long_COVID	1_percent	0					
		5_percent	0.03886	0.11218	0.34643	0.72902	-0.18100	0.25872
		10_percent	0.02336	0.11634	0.20078	0.84087	-0.20467	0.25139
		20_percent	0.14352	0.11433	1.25530	0.20937	-0.08057	0.36762
	Green_pass	Not_needed	0					
		Needed	-0.05709	0.08159	-0.69976	0.48407	-0.21700	0.10282
	Vaccine_mandate	No	0					
		Age_50+_fine_100€	-0.11236	0.10136	-1.10849	0.26765	-0.31102	0.08631
		Age_18+_fine_1500€	-0.15528	0.10107	-1.53637	0.12445	-0.35338	0.04281
1 or 2	Consensus	Consensus_scientists	0					
		Consensus_physicians	-0.03827	0.15135	-0.25284	0.80039	-0.33491	0.25838
		Dissensus_scientists	0.03002	0.15661	0.19168	0.84799	-0.27694	0.33698
		Dissensus_physicians	0.19407	0.14876	1.30458	0.19204	-0.09750	0.48564
	Celebrity	Refuses_vaccine	0					
		Endorses_vaccine	0.07579	0.15056	0.50336	0.61471	-0.21931	0.37089
		Waits_for_new_vaccine	-0.01511	0.15103	-0.10003	0.92032	-0.31113	0.28091
		Infected_regrets_hesitancy	0.23430	0.14959	1.56622	0.11730	-0.05890	0.52750
	Long_COVID	1_percent	0					
		5_percent	0.05766	0.14387	0.40079	0.68857	-0.22432	0.33964
		10_percent	0.03095	0.14479	0.21373	0.83076	-0.25283	0.31472
		20_percent	0.10209	0.14455	0.70630	0.48000	-0.18121	0.38540
	Green_pass	Not_needed	0					
		Needed	-0.04393	0.10937	-0.40168	0.68792	-0.25829	0.17043
	Vaccine_mandate	No	0					
		Age_50+_fine_100€	-0.19357	0.12302	-1.57344	0.11562	-0.43469	0.04755
		Age_18+_fine_1500€	-0.08558	0.13945	-0.61368	0.53943	-0.35888	0.18773
≥3	Consensus	Consensus_scientists	0					
		Consensus_physicians	0.08556	0.06519	1.31250	0.18935	-0.04221	0.21332
		Dissensus_scientists	-0.13272	0.06646	-1.99705	**0.04582**	-0.26297	-0.00246
		Dissensus_physicians	-0.16135	0.06719	-2.40158	**0.01632**	-0.29303	-0.02967
	Celebrity	Refuses_vaccine	0					

Sub-group	Attribute	Levels	Est.	SE	z	p	95% Confidence interval	
							lower	upper
		Endorses_vaccine	0.6919	0.06648	2.54506	**0.01093**	0.03890	0.29948
		Waits_for_new_vaccine	0.00866	0.06685	0.12957	0.89691	-0.12235	0.13968
		Infected_regrets_hesitancy	0.13402	0.06606	2.02869	**0.04249**	0.00454	0.26350
	Long_COVID	1_percent	0					
		5_percent	0.00656	0.06941	0.09451	0.92470	-0.12948	0.14260
		10_percent	-0.02200	0.06681	-0.32923	0.74198	-0.15295	0.10896
		20_percent	0.01045	0.06858	0.15230	0.87895	-0.12398	0.14487
	Green_pass	Not_needed	0					
		Needed	0.06838	0.04569	1.49643	0.13454	-0.02118	0.15794
	Vaccine_mandate	No	0					
		Age_50+_fine_100€	0.01704	0.05943	0.28673	0.77432	-0.0994	0.13351
		Age_18+_fine_1500€	0.07564	0.05955	1.27020	0.20401	-0.04108	0.19237

Notes: Estimates are Average Marginal Component Effects (AMCEs).

Supplemental File 13. Effects on the evaluation of the campaign by gender (experiment 1)

We calculated Average Marginal Component Effects (AMCEs). Data are presented as AMCE estimates +/- 95% confidence intervals. Exact p-values are shown in column 7. The Bonferroni corrected significance level is 0.0031. Significant p-values are marked with (in bold and with a *) and without Bonferroni correction (in bold). Gender differences are marked in grey; p-values were marked if women had a significant p-values and men had not or vice versa.

Sub-group	Attribute	Levels	Est.	SE	z	p	95% Confidence interval	
							lower	upper
Female	Virus_variant	No_change	0					
		Escalation	0.02137	0.01085	1.96871	**0.04899**	0.00009	0.04264
		Decline	-0.04068	0.01068	-3.80995	**0.00014***	-0.06161	-0.01975
	Vaccines	mRNA_only	0					
		mRNA+Inactive	0.01509	0.00897	1.68299	0.09238	-0.00248	0.03266
	Omicron_adapted	Not_adapted	0					
		Adapted	0.07986	0.00887	9.00700	**<0.00001***	0.06248	0.09723
	Incentives	Free	0					
		20_Euro_fee	-0.18190	0.01252	-14.53134	**<0.00001***	-0.20643	-0.15736
		500_Euro_cash	-0.01199	0.01304	-0.91945	0.35786	-0.03754	0.01356
		500_Euro_voucher	-0.02006	0.01287	-1.55919	0.11895	-0.04528	0.00516
	Motivation	Risk_re-infection	0					
		Risk_unable_to_work	0.02198	0.01751	1.25546	0.20931	-0.01233	0.05629
		Risk_severe_disease	0.04798	0.01747	2.74575	**0.00604**	0.01373	0.08223
		Community_spirit	0.03850	0.01767	2.17916	**0.02932**	0.00387	0.07313
		Protect_friends	0.05822	0.01737	3.35138	**0.00080***	0.02417	0.09227
		Self-efficacy	0.04661	0.01731	2.69281	**0.00709**	0.01269	0.08054
		Risk_lockdown	0.02875	0.01736	1.65620	0.09768	-0.00527	0.06277
		Protect_health_system	0.05406	0.01748	3.09222	**0.00199***	0.01980	0.08833
Male	Virus_variant	No_change	0					
		Escalation	0.00531	0.01115	0.47619	0.63394	-0.01654	0.02715
		Decline	-0.01977	0.01107	-1.78595	0.07411	-0.04148	0.00193
	Vaccines	mRNA_only	0					
		mRNA+Inactive	0.01777	0.00899	1.97608	**0.04815**	0.00014	0.03540
	Omicron_adapted	Not_adapted	0.00000					
		Adapted	0.07452	0.00912	8.16905	**<0.00001***	0.05664	0.09240
	Incentives	Free	0					
		20_Euro_fee	-0.14660	0.01258	-11.65099	**<0.00001***	-0.17126	-0.12194
		500_Euro_cash	0.04265	0.01276	3.34149	**0.00083***	0.01763	0.06767
		500_Euro_voucher	0.01695	0.01289	1.31559	0.18831	-0.00830	0.04221
	Motivation	Risk_re-infection	0.00000					
		Risk_unable_to_work	-0.01259	0.01813	-0.69479	0.48719	-0.04812	0.02293
		Risk_severe_disease	0.01963	0.01821	1.07829	0.28091	-0.01605	0.05532
		Community_spirit	0.03416	0.01814	1.88291	0.05971	-0.00140	0.06973
		Protect_friends	0.01954	0.01837	1.06335	0.28762	-0.01647	0.05555
		Self-efficacy	0.02807	0.01834	1.53058	0.12587	-0.00787	0.06401
		Risk_lockdown	0.03560	0.01842	1.93292	0.05325	-0.00050	0.07170
		Protect_health_system	0.03868	0.01828	2.11583	**0.03436**	0.00285	0.07452

Notes: Estimates are Average Marginal Component Effects (AMCEs).

Supplemental File 14. Effects on the likelihood to get vaccinated by gender (experiment 1)

We calculated Average Marginal Component Effects (AMCEs). Data are presented as AMCE estimates +/- 95% confidence intervals. Exact p-values are shown in column 7. The Bonferroni corrected significance level is 0.0031. Significant p-values are marked with (in bold and with a *) and without Bonferroni correction (in bold). Gender differences are marked in grey; p-values were marked if women had a significant p-values and men had not or vice versa.

Sub-group	Attribute	Levels	Est.	SE	z	p	95% Confidence interval	
							lower	upper
Female	Virus_variant	No_change	0					
		Escalation	0.07057	0.07441	0.94842	0.34291	-0.07527	0.21641
		Decline	-0.10785	0.07466	-1.44455	0.14858	-0.25419	0.03848
	Vaccines	mRNA_only	0					
		mRNA+Inactive	-0.10597	0.06223	-1.70277	0.08861	-0.22795	0.01601
	Omicron_adapted	Not_adapted	0					
		Adapted	0.15947	0.06370	2.50342	**0.01230**	0.03462	0.28433
	Incentives	Free	0					
		20_Euro_fee	-0.52585	0.08927	-5.89080	**<0.00001***	-0.70081	-0.35089
		500_Euro_cash	0.10172	0.08832	1.15178	0.24941	-0.07138	0.27482
		500_Euro_voucher	0.19646	0.08883	2.21173	**0.02699**	0.02236	0.37056
	Motivation	Risk_re-infection	0					
		Risk_unable_to_work	0.01466	0.12359	0.11863	0.90557	-0.22757	0.25690
		Risk_severe_disease	0.18634	0.12471	1.49412	0.13514	-0.05810	0.43078
		Community_spirit	0.07099	0.12449	0.57029	0.56848	-0.17300	0.31499
		Protect_friends	-0.15984	0.12556	-1.27303	0.20301	-0.40594	0.08625
		Self-efficacy	0.03390	0.12377	0.27389	0.78417	-0.20868	0.27648
		Risk_lockdown	0.10532	0.12350	0.85280	0.39377	-0.13674	0.34739
		Protect_health_system	0.05708	0.12725	0.44860	0.65372	-0.19232	0.30649
Male	Virus_variant	No_change	0					
		Escalation	-0.00173	0.07333	-0.02363	0.98115	-0.14546	0.14200
		Decline	-0.10331	0.07143	-1.44636	0.14808	-0.24331	0.03669
	Vaccines	mRNA_only	0					
		mRNA+Inactive	0.05616	0.05994	0.93688	0.34882	-0.06133	0.17365
	Omicron_adapted	Not_adapted	0					
		Adapted	0.22458	0.06101	3.68087	**0.00023***	0.10499	0.34416
	Incentives	Free	0					
		20_Euro_fee	-0.62922	0.08649	-7.27519	**<0.00001***	-0.79873	-0.45970
		500_Euro_cash	0.29561	0.08643	3.42004	**0.00063***	0.12620	0.46502
		500_Euro_voucher	0.31686	0.08766	3.61472	**0.00030***	0.14505	0.48867
	Motivation	Risk_re-infection	0					
		Risk_unable_to_work	-0.14664	0.11707	-1.25257	0.21036	-0.37609	0.08281
		Risk_severe_disease	0.02961	0.11852	0.24980	0.80274	-0.20269	0.26191
		Community_spirit	0.06891	0.11747	0.58663	0.55745	-0.16133	0.29916
		Protect_friends	-0.05876	0.11891	-0.49412	0.62122	-0.29183	0.17431
		Self-efficacy	-0.04296	0.11878	-0.36169	0.71759	-0.27577	0.18984
		Risk_lockdown	0.00784	0.11548	0.06788	0.94588	-0.21849	0.23417
		Protect_health_system	0.06912	0.11743	0.58865	0.55610	-0.16103	0.29928

Notes: Estimates are Average Marginal Component Effects (AMCEs).

Supplemental File 15. Effects on trust in the vaccine by gender (experiment 2)

We calculated Average Marginal Component Effects (AMCEs). Data are presented as AMCE estimates +/- 95% confidence intervals. Exact p-values are shown in column 7. The Bonferroni corrected significance level is 0.0041. Significant p-values are marked with (in bold and with a *) and without Bonferroni correction (in bold). Gender differences are marked in grey; p-values were marked if women had a significant p-values and men had not or vice versa.

Sub-group	Attribute	Levels	Est.	SE	z	p	95% Confidence interval	
							lower	upper
Female	Consensus	Consensus_scientists	0					
		Consensus_physicians	-0.02644	0.01245	-2.12356	**0.03371**	-0.05084	-0.00204
		Dissensus_scientists	-0.05795	0.01266	-4.57840	**<0.00001***	-0.08276	-0.03314
		Dissensus_physicians	-0.07805	0.01282	-6.08724	**<0.00001***	-0.10318	-0.05292
	Celebrity	Refuses_vaccine	0					
		Endorses_vaccine	0.04252	0.01279	3.32598	**0.00088***	0.01747	0.06758
		Waits_for_new_vaccine	0.00243	0.01237	0.19618	0.84447	-0.02181	0.02666
		Infected_regrets_hesitancy	0.04892	0.01239	3.94724	**0.00008***	0.02463	0.07320
	Long_COVID	1_percent	0					
		5_percent	0.02182	0.01248	1.74862	0.08036	-0.00264	0.04627
		10_percent	0.00415	0.01229	0.33736	0.73584	-0.01995	0.02824
		20_percent	0.03086	0.01239	2.49131	**0.01273**	0.00658	0.05514
	Green_pass	Not_needed	0					
		Needed	0.01060	0.00924	1.14696	0.25140	-0.00751	0.02870
	Vaccine_mandate	No	0					
		Age_50+_fine_100€	-0.09652	0.01112	-8.67708	**<0.00001***	-0.11832	-0.07472
		Age_18+_fine_1500€	-0.13998	0.01140	-12.28409	**<0.00001***	-0.16232	-0.11765
Male	Consensus	Consensus_scientists	0					
		Consensus_physicians	-0.01813	0.01319	-1.37418	0.16939	-0.04399	0.00773
		Dissensus_scientists	-0.06696	0.01281	-5.22830	**<0.00001***	-0.09206	-0.04186
		Dissensus_physicians	-0.06220	0.01305	-4.76764	**<0.00001***	-0.08778	-0.03663
	Celebrity	Refuses_vaccine	0					
		Endorses_vaccine	0.06312	0.01309	4.82131	**<0.00001***	0.03746	0.08878
		Waits_for_new_vaccine	0.04412	0.01274	3.46348	**0.00053***	0.01915	0.06909
		Infected_regrets_hesitancy	0.06689	0.01291	5.17995	**<0.00001***	0.04158	0.09220
	Long_COVID	1_percent	0					
		5_percent	0.01478	0.01281	1.15354	0.24869	-0.01033	0.03990
		10_percent	0.00228	0.01271	0.17951	0.85754	-0.02263	0.02719
		20_percent	0.01725	0.01292	1.33566	0.18166	-0.00806	0.04256
	Green_pass	Not_needed	0					
		Needed	0.00140	0.00905	0.15510	0.87674	-0.01633	0.01914
	Vaccine_mandate	No	0					
		Age_50+_fine_100€	-0.06151	0.01140	-5.39607	**<0.00001***	-0.08386	-0.03917
		Age_18+_fine_1500€	-0.12031	0.01176	-10.23147	**<0.00001***	-0.14335	-0.09726

Notes: Estimates are Average Marginal Component Effects (AMCEs).

Supplemental File 16. Effects on the likelihood to get vaccinated by gender (experiment 2)

We calculated Average Marginal Component Effects (AMCEs). Data are presented as AMCE estimates +/- 95% confidence intervals. Exact p-values are shown in column 7. The Bonferroni corrected significance level is 0.0041. Significant p-values are marked with (in bold and with a *) and without Bonferroni correction (in bold). Gender differences are marked in grey; p-values were marked if women had a significant p-values and men had not or vice versa.

Sub-group	Attribute	Levels	Est.	SE	z	P	95% Confidence interval	
							lower	upper
AT	Consensus	Consensus_scientists	0					
		Consensus_physicians	0.03225	0.08582	0.37578	0.70708	-0.13595	0.20045
		Dissensus_scientists	-0.12908	0.08888	-1.45221	0.14644	-0.30329	0.04513
		Dissensus_physicians	-0.14308	0.08654	-1.65329	0.09827	-0.31269	0.02654
	Celebrity	Refuses_vaccine	0					
		Endorses_vaccine	0.19234	0.08813	2.18246	**0.02908**	0.01961	0.36506
		Waits_for_new_vaccine	0.02127	0.08544	0.24890	0.80344	-0.14619	0.18872
		Infected_regrets_hesitancy	0.17766	0.08752	2.02993	**0.04236**	0.00612	0.34919
	Long_COVID	1_percent	0					
		5_percent	0.10257	0.08824	1.16243	0.24506	-0.07038	0.27552
		10_percent	0.01848	0.08828	0.20936	0.83417	-0.15455	0.19152
		20_percent	0.15102	0.08711	1.73359	0.08299	-0.01972	0.32176
	Green_pass	Not_needed	0					
		Needed	0.11853	0.06193	1.91400	0.05562	-0.00285	0.23991
	Vaccine_mandate	No	0					
		Age_50+_fine_100€	0.08118	0.07333	1.10701	0.26829	-0.06255	0.22490
		Age_18+_fine_1500€	0.12253	0.07627	1.60654	0.10816	-0.02695	0.27201
Male	Consensus	Consensus_scientists	0					
		Consensus_physicians	0.19873	0.08394	2.36750	**0.01791**	0.03421	0.36325
		Dissensus_scientists	0.08589	0.08292	1.03579	0.30030	-0.07663	0.24840
		Dissensus_physicians	0.06472	0.08640	0.74904	0.45383	-0.10463	0.23406
	Celebrity	Refuses_vaccine	0					
		Endorses_vaccine	0.05360	0.08276	0.64771	0.51717	-0.10860	0.21581
		Waits_for_new_vaccine	0.03631	0.08323	0.43627	0.66264	-0.12682	0.19944
		Infected_regrets_hesitancy	0.10074	0.08237	1.22302	0.22132	-0.06070	0.26219
	Long_COVID	1_percent	0					
		5_percent	-0.03209	0.08293	-0.38692	0.69882	-0.19463	0.13046
		10_percent	-0.02008	0.08140	-0.24667	0.80516	-0.17962	0.13946
		20_percent	0.02357	0.08338	0.28265	0.77745	-0.13985	0.18698
	Green_pass	Not_needed	0					
		Needed	-0.05099	0.05829	-0.87470	0.38174	-0.16524	0.06326
	Vaccine_mandate	No	0					
		Age_50+_fine_100€	-0.10624	0.07463	-1.42362	0.15456	-0.25252	0.04003
		Age_18+_fine_1500€	-0.08438	0.07415	-1.13799	0.25512	-0.22971	0.06095

Notes: Estimates are Average Marginal Component Effects (AMCEs).

Supplemental File 17. STROBE Statement

Checklist of items that should be included in reports of observational studies.

	Item No	Recommendation	Implementation
**Title and abstract**	1	(*a*) Indicate the study's design with a commonly used term in the title or the abstract	The study's design is included in the abstract.
		(*b*) Provide in the abstract an informative and balanced summary of what was done and what was found	The abstract includes background, method, results and conclusions.
**Introduction**			
Background/rationale	2	Explain the scientific background and rationale for the investigation being reported	Explained in the introduction.
Objectives	3	State specific objectives, including any prespecified hypotheses	Explained in the introduction.
**Methods**			
Study design	4	Present key elements of study design early in the paper	Described in the fifth paragraph of the main text.
Setting	5	Describe the setting, locations, and relevant dates, including periods of recruitment, exposure, follow-up, and data collection	Explained in the introduction (last paragraph) and the methods section.
Participants	6	(*a*) *Cohort study*—Give the eligibility criteria, and the sources and methods of selection of participants. Describe methods of follow-up<br>*Case-control study*—Give the eligibility criteria, and the sources and methods of case ascertainment and control selection. Give the rationale for the choice of cases and controls<br>*Cross-sectional study*—Give the eligibility criteria, and the sources and methods of selection of participants	Explained in the introduction (last paragraph) and the methods section.
		(*b*) *Cohort study*—For matched studies, give matching criteria and number of exposed and unexposed<br>*Case-control study*—For matched studies, give matching criteria and the number of controls per case	NA
Variables	7	Clearly define all outcomes, exposures, predictors, potential confounders, and effect modifiers. Give diagnostic criteria, if applicable	Included in the introduction and the methods section where we give details about the variables collected and the procedures and exact wording of the attributes and levels of

	Item No	Recommendation	Implementation
			the conjoint experiments.
Data sources/ measurement	8*	For each variable of interest, give sources of data and details of methods of assessment (measurement). Describe comparability of assessment methods if there is more than one group	Described in the methods section and the appendix.
Bias	9	Describe any efforts to address potential sources of bias	Explained in the introduction and the methods section.
Study size	10	Explain how the study size was arrived at	Described in the methods section.
Quantitative variables	11	Explain how quantitative variables were handled in the analyses. If applicable, describe which groupings were chosen and why	Described in the methods section.
Statistical methods	12	(*a*) Describe all statistical methods, including those used to control for confounding	Described in the methods section.
		(*b*) Describe any methods used to examine subgroups and interactions	Described in the methods section.
		(*c*) Explain how missing data were addressed	Described in the methods section.
		(*d*) *Cohort study*—If applicable, explain how loss to follow-up was addressed<br>*Case-control study*—If applicable, explain how matching of cases and controls was addressed<br>*Cross-sectional study*—If applicable, describe analytical methods taking account of sampling strategy	NA
		(*e*) Describe any sensitivity analyses	NA

Continued on next page

Results

Participants	13*	(a) Report numbers of individuals at each stage of study—eg numbers potentially eligible, examined for eligibility, confirmed eligible, included in the study, completing follow-up, and analysed	Described in the results section and the appendix (Supplemental File 1).
		(b) Give reasons for non-participation at each stage	NA
		(c) Consider use of a flow diagram	NA
Descriptive data	14*	(a) Give characteristics of study participants (eg demographic, clinical, social) and information on exposures and potential confounders	Described in the results section and the Extended Data Figure 1.
		(b) Indicate number of participants with missing data for each variable of interest	We describe in the methods section how we dealt with missing data. Extended Data Figures 1 and 2 report frequencies of responses and missing data.
		(c) *Cohort study*—Summarise follow-up time (eg, average and total amount)	NA
Outcome data	15*	*Cohort study*—Report numbers of outcome events or summary measures over time	NA
		Case-control study—Report numbers in each exposure category, or summary measures of exposure	NA
		Cross-sectional study—Report numbers of outcome events or summary measures	Reported in the results section, as well as in the appendix.
Main results	16	(*a*) Give unadjusted estimates and, if applicable, confounder-adjusted estimates and their precision (eg, 95% confidence interval). Make clear which confounders were adjusted for and why they were included	Reported in the appendix (Supplemental Files 5 to 16).
		(*b*) Report category boundaries when continuous variables were categorized	NA
		(*c*) If relevant, consider translating estimates of relative risk into absolute risk for a meaningful time period	NA
Other analyses	17	Report other analyses done—eg analyses of subgroups and interactions, and sensitivity analyses	Methods were used to analyse the data, including descriptive statistics, the estimation of Average Conditional Marginal Effects (ACMEs), and graphical depictions (e.g. boxplots). Findings are presented in the results section, the Extended Data Figures and the appendix.

**Discussion**			
Key results	18	Summarise key results with reference to study objectives	Described in the discussion.
Limitations	19	Discuss limitations of the study, taking into account sources of potential bias or imprecision. Discuss both direction and magnitude of any potential bias	Described in the discussion.
Interpretation	20	Give a cautious overall interpretation of results considering objectives, limitations, multiplicity of analyses, results from similar studies, and other relevant evidence	Described in the discussion.
Generalisability	21	Discuss the generalisability (external validity) of the study results	Described in the discussion.
**Other information**			
Funding	22	Give the source of funding and the role of the funders for the present study and, if applicable, for the original study on which the present article is based	Included in the acknowledgement section.

*Give information separately for cases and controls in case-control studies and, if applicable, for exposed and unexposed groups in cohorts and cross-sectional studies.

Note: An Explanation and Elaboration article discusses each checklist item and gives methodological background and published examples of transparent reporting. The STROBE checklist is best used in conjunction with this article (freely available on the Web sites of PLoS Medicine at http://www.plosmedicine.org/, Annals of Internal Medicine at http://www.annals.org/, and Epidemiology at http://www.epidem.com/). Information on the STROBE Initiative is available at www.strobe-statement.org.
