# Supplementary material for: Determinants of COVID-19 vaccine fatigue
Source: Nat Med. 2023 Mar 27;29(5):1164–71. doi: 10.1038/s41591-023-02282-y (PMC10202806; doi:10.1038/s41591-023-02282-y)
Supplement: Supplementary file 2 — Reporting Summary [file 41591_2023_2282_MOESM2_ESM.pdf]

nature portfolio

Corresponding author(s): Stamm, Partheymueller, Mosor, Ritschl, Kritzinger, Alunno, Eberl

Last updated by author(s): 27/02/23

Reporting Summary

Nature Portfolio wishes to improve the reproducibility of the work that we publish. This form provides structure for consistency and transparency in reporting. For further information on Nature Portfolio policies, see our Editorial Policies and the Editorial Policy Checklist.

Statistics

For all statistical analyses, confirm that the following items are present in the figure legend, table legend, main text, or Methods section.

n/a	Confirmed	
☐	☒	The exact sample size (*n*) for each experimental group/condition, given as a discrete number and unit of measurement
☐	☒	A statement on whether measurements were taken from distinct samples or whether the same sample was measured repeatedly
☐	☒	The statistical test(s) used AND whether they are one- or two-sided<br>*Only common tests should be described solely by name; describe more complex techniques in the Methods section.*
☐	☒	A description of all covariates tested
☐	☒	A description of any assumptions or corrections, such as tests of normality and adjustment for multiple comparisons
☐	☒	A full description of the statistical parameters including central tendency (e.g. means) or other basic estimates (e.g. regression coefficient) AND variation (e.g. standard deviation) or associated estimates of uncertainty (e.g. confidence intervals)
☐	☒	For null hypothesis testing, the test statistic (e.g. F, t, r) with confidence intervals, effect sizes, degrees of freedom and P value noted<br>*Give P values as exact values whenever suitable.*
☒	☐	For Bayesian analysis, information on the choice of priors and Markov chain Monte Carlo settings
☒	☐	For hierarchical and complex designs, identification of the appropriate level for tests and full reporting of outcomes
☒	☐	Estimates of effect sizes (e.g. Cohen's *d*, Pearson's *r*), indicating how they were calculated

Our web collection on statistics for biologists contains articles on many of the points above.

Software and code

Policy information about availability of computer code

Data collection	We used a software developed for Marketagent's commercial online access panel (certified under ISO 20252) for data collection. Marketagent's software is an own development without version designation.
Data analysis	We used the R version 4.1.2 (2021-11-01) -- "Bird Hippie" for data analysis. We published the analytic code associated with this study at https://doi.org/10.7910/DVN/3R2CMT (Harvard Dataverse).

For manuscripts utilizing custom algorithms or software that are central to the research but not yet described in published literature, software must be made available to editors and reviewers. We strongly encourage code deposition in a community repository (e.g. GitHub). See the Nature Portfolio guidelines for submitting code & software for further information.

Data

Policy information about availability of data

All manuscripts must include a data availability statement. This statement should provide the following information, where applicable:

- Accession codes, unique identifiers, or web links for publicly available datasets
- A description of any restrictions on data availability
- For clinical datasets or third party data, please ensure that the statement adheres to our policy

The raw data generated in this study are publicly available at https://doi.org/10.7910/DVN/3R2CMT (Harvard Dataverse).

Human research participants

Policy information about studies involving human research participants and Sex and Gender in Research.

Reporting on sex and gender	Participants self-reported their gender.
Population characteristics	The study sample matched the target distributions regarding gender, age groups, regions, and education of the resident population (> 14 years) in Austria and Italy. Target quotas and the actual absolute and relative frequencies regarding gender, age groups, education and region of residency are shown in Supplemental File 1. We chose a sample that matched the characteristics of the general population to ensure generalizablility.
Recruitment	We used population-representative quotas to recruit the respondents from the commercial online access panel by Marketagent GmbH (certified under ISO 20252), encompassing more than 135,000 registered panelists in Austria and 87,000 in Italy. The target population in each country were residents aged 14+ years. Please note that from the age of 14, adolescents in Austria and Italy can decide for themselves, without their parents' consent, whether they want to be vaccinated or not. The survey was initially opened for 61,503 Austrians and 73,077 Italians and closed after the target quotas were reached in each country. Eventually, 3,187 participants from Austria and 3,170 participants from Italy took part in the study after providing informed consent.<br>The participation rate was overall similar to comparable studies. The quota targets and actual values in the sample are shown in Supplemental File 1. Please note that, although we aimed to make the sample structure match as closely as possible to the targets, some subgroups of the population remain hard to reach for online surveys (e.g. very high age, language minorities).
Ethics oversight	All survey participants provided informed consent to the survey company that carried out the fieldwork. Only anonymized data was received and analyzed. Research ethics approval for this study was not required according to institutional and national guidelines, such as the Medical Devices Act of Austria.

Note that full information on the approval of the study protocol must also be provided in the manuscript.

Field-specific reporting

Please select the one below that is the best fit for your research. If you are not sure, read the appropriate sections before making your selection.

☐ Life sciences ☒ Behavioural & social sciences ☐ Ecological, evolutionary & environmental sciences

For a reference copy of the document with all sections, see nature.com/documents/nr-reporting-summary-flat.pdf

Behavioural & social sciences study design

All studies must disclose on these points even when the disclosure is negative.

Study description	Two conjoint experiments embedded in a cross-sectional online survey
Research sample	The study sample matched the target distributions regarding gender, age groups, regions, and education of the general population in Austria and Italy.
Sampling strategy	Participants were recruited based on quotas for the respective resident population (> 14 years) from the commercial online access panel by Marketagent GmbH (certified under ISO 20252).
Data collection	Marketagent GmbH used their online access panel certified under ISO 20252. The participants viewed the case vignettes and filled the survey questions in alone with no researcher being resent.
Timing	From July 19th to August 8th, 2022
Data exclusions	Respondents were required to answer the questions of the conjoint experiments in order to participate in the study. For the binary choice questions respondents were instructed to choose spontaneously one of the two options if they felt indifferent. Therefore, there are no missing values on our outcome variable and all participants were included in the analysis. The questions that were included in the survey for descriptive purposes such as sociodemographics, measures of trust and emotions offered opt-out options ("Don't know", "No answer"). When calculating descriptive statistics, such responses were excluded from the analysis. We explain this in the methods section.
Non-participation	A total of 61,503 persons were invited in Austria and 73,077 in Italy. 5% of the Austrian and 4% of the Italian participants who were initially approached participated. However, please note, that after the calculated number of cases stratified according to the quotas in both countries was reached, the survey was closed. The participation rate was overall similar to comparable studies. We explain this in the methods section.
Randomization	In the two conjoint experiments, we varied attribute levels randomly to assess which components of a multidimensional treatment

Randomization	would be influential. The complete list of attributes and their levels as well as the wording of the prompt and follow-up questions are available in Supplemental Files 3 and 4.

Reporting for specific materials, systems and methods

We require information from authors about some types of materials, experimental systems and methods used in many studies. Here, indicate whether each material, system or method listed is relevant to your study. If you are not sure if a list item applies to your research, read the appropriate section before selecting a response.

Materials & experimental systems

n/a	Involved in the study
☒	☐ Antibodies
☒	☐ Eukaryotic cell lines
☒	☐ Palaeontology and archaeology
☒	☐ Animals and other organisms
☒	☐ Clinical data
☒	☐ Dual use research of concern

Methods

n/a	Involved in the study
☒	☐ ChIP-seq
☒	☐ Flow cytometry
☒	☐ MRI-based neuroimaging
